# Saturated Cannabinoids: Update on Synthesis Strategies and Biological Studies of These Emerging Cannabinoid Analogs

**DOI:** 10.3390/molecules28176434

**Published:** 2023-09-04

**Authors:** Maite L. Docampo-Palacios, Giovanni A. Ramirez, Tesfay T. Tesfatsion, Alex Okhovat, Monica Pittiglio, Kyle P. Ray, Westley Cruces

**Affiliations:** Colorado Chromatography Labs, 10505 S. Progress Way, Unit 105, Parker, CO 80134, USA; giovanni@coloradochromatography.com (G.A.R.); tesfay@coloradochromatography.com (T.T.T.); alex.o@coloradochromatography.com (A.O.); monica@sunflowerwellness.us (M.P.); kyle@coloradochromatography.com (K.P.R.)

**Keywords:** cannabinoids, hydrogenation, hexahydrocannabinol, cannabilactones, quinones, CB1 receptor, CB2 receptor, GPCR

## Abstract

Natural and non-natural hexahydrocannabinols (HHC) were first described in 1940 by Adam and in late 2021 arose on the drug market in the United States and in some European countries. A background on the discovery, synthesis, and pharmacology studies of hydrogenated and saturated cannabinoids is described. This is harmonized with a summary and comparison of the cannabinoid receptor affinities of various classical, hybrid, and non-classical saturated cannabinoids. A discussion of structure–activity relationships with the four different pharmacophores found in the cannabinoid scaffold is added to this review. According to laboratory studies in vitro, and in several animal species in vivo, HHC is reported to have broadly similar effects to Δ9-tetrahydrocannabinol (Δ9-THC), the main psychoactive substance in cannabis, as demonstrated both in vitro and in several animal species in vivo. However, the effects of HHC treatment have not been studied in humans, and thus a biological profile has not been established.

## 1. Introduction

Cannabis and cannabis substituents have been used in medicine within the United States for centuries and were first described in the United States Pharmacopeia in late 1850 [1]. Due to legal ramifications and political duress, cannabis was dropped from the United States Pharmacopeia in the 1940s and labeled a controlled substance in the 1970s. These bureaucratic changes have limited advancements within the field of cannabinoid chemistry [2]. The first cannabinoid was not elucidated until the 1940s, when cannabidiol (CBD) was identified, followed by cannabinol (CBN) [3]. As cannabinoid research becomes accessible again, novel and rare cannabinoids have been elucidated through modern analytical techniques, garnering attention, and popularity. However, knowledge about these cannabinoids remains limited to non-existent. Cannabinoid research as a whole has primarily focused on the safety and efficacy of CBD and THC (tetrahydrocannabinol) for specific ailments and has largely ignored the hundreds of other currently identified cannabinoids that *Cannabis sativa* biosynthesizes in various concentrations [1,2,3,4]. The primary focus of cannabinoid chemistry and the multitude of studies that have been performed are mostly on CBD, and THC, evaluating their safety and effects on certain ailments including but not limited to inflammation and anti-proliferative/pro-apoptotic effects within the body [5].

Of the limited studies on cannabinoid derivatives, minute amounts of data are produced on saturated cannabinoid derivatives [6,7]. Several studies that have been published focused on hydroxyl derivatives of hydrogenated THC such as 9-Nor-9β-hydroxyhexahydrocannabinol (9-Nor-9β-HHC), 9-Hydroxyhexahydrocannabinol (9-OH-HHC), or 11-Hydroxyhexahydrocannabinol (11-OH-HHC and 7-OH-HHC), which are identified as metabolites of THC. Commonly confused with HHC (Hexahydrocannabinol) that is in research and consumer markets, due to the nomenclature used, no detailed information is focused on the hydrogenated derivatives of various cannabinoids such as CBD, THC, THCV (Tetrahydrocannabivarin), and CBDV (Cannabidivarin). As the popularity of cannabinoids skyrockets, so does the need for markets to continually update with derivatives that are homologous to THC, CBD, CBDV, and THCV.

Since its discovery in 1940, through catalytic hydrogenation of THC and cannabinoid derivatives, hydrogenated cannabinoids have been synthesized; only H_4_CBD and HHC have been of interest as they are the hydrogenated scaffolds of THC and CBD [8].

The rediscovery of these hydrogenated derivatives is pushing into the medicinal properties that they might share with their parental counterparts. In an earlier study produced by Gallily et al. in 2006 [9], hydrogenated cannabinoid derivatives of CBD and the CBD-DM (cannabidiol–dimethylheptyl) scaffolds, which included a mixture of H_4_CBD diastereomers, determined that diastereomers of H_4_CBD bound to the CB_1_ receptor with great affinity, and the anti-inflammatory capacity of H_4_CBD was reported [9]. While minute preliminary studies on the mechanism and the binding affinities of H_4_CBD have been produced, no in-depth toxicological profile has been created for H_4_CBD and HHC, aside from pre-clinical in vitro data that have been published to determine general consumption safety and characterization [10,11].

Against this backdrop, we embark on a comprehensive and critical review, drawing upon meticulously selected published research obtained from esteemed sources such as PubMed, Scopus databases, official international organizations’ websites, and others covering from 1940 to 2023.

Our intention is to shed light on the present clinical evidence concerning not only hydrogenated derivatives of THC and CBD but also the other captivating, saturated cannabinoids discovered within the *Cannabis sativa* plant. Additionally, we aim to provide critical insights into the sufficiency of this evidence in supporting their synthesis, characterization, and possible utilization as medicinal substances. By undertaking this endeavor, we hope to contribute to the broader understanding of saturated cannabinoids and their potential therapeutic applications, while addressing the need for further research in this promising field.

## 2. Saturated Tricyclic Hexahydrocannabinol Homologs

Since its discovery in 1964, tetrahydrocannabinol (THC) and related analogs such as cannabidiol (CBD) and natural and non-natural saturated cannabinoids have caught the attention of research groups all over the world [12,13,14,15]. Hexahydrocannabinol (HHC) is a newer cannabinoid to hit the cannabis consumer market, but it is not exactly a new cannabinoid. HHC was discovered in 1944 by the American chemist Roger Adams [8] while exploring with the hydrogenation reaction with the THC molecule in marijuana.

**Figure 1:**
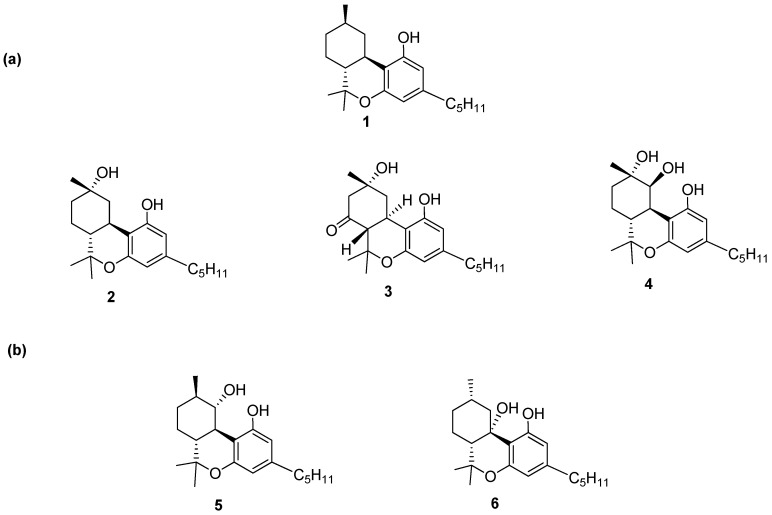
Hexahydrocannabinols isolated from *Cannabis sativa* plants. Minor oxygenated cannabinoids have been identified as trace components in *Cannabis sativa* plants. They are formed as degenerative byproducts as the THC breaks down. In (**a**) (**1**) (9*R*)-6,6,9-trimethyl-3-pentyl-6a,7,8,9,10,10a-hexahydro-6*H*-benzo[c]chromen-1-ol, (6a*R*,9*S*,10a*R*)-6,6,9-trimethyl-3-pentyl-6a,7,8,9,10,10a-hexahydro-6*H*-benzo[c]chromene-1,9-diol (**2**), (6a*R*,9*R*,10a*R*)-1,9-dihydroxy-6,6,9-trimethyl-3-pentyl-8,9,10,10a-tetrahydro-6*H*-benzo[c]chromen-7(6a*H*)-one (**3**), (6a*R*,9*S*,10*S*,10a*R*)-6,6,9-trimethyl-3-pentyl-6a,7,8,9,10,10a-hexahydro-6*H*-benzo[c]chromene-1,9,10-triol. In (**b**), (6a*R*,9*R*,10*S*,10a*R*)-6,6,9-trimethyl-3-pentyl-6a,7,8,9,10,10a-hexahydro-6*H*-benzo[c]chromene-1,10-diol (**5**), and (6a*R*,9*S*,10a*S*)-6,6,9-trimethyl-3-pentyl-6a,7,8,9,10,10a-hexahydro-6*H*-benzo[c]chromene-1,10a-diol.

Also, (9*R*)-6,6,9-trimethyl-3-pentyl-6a,7,8,9,10,10a-hexahydro-6*H*-benzo[c]chromen-1-ol and other minor oxygenated cannabinoids have been identified as trace components in *Cannabis sativa* plants. They are formed as degenerative byproducts as the THC breaks down [16] (Figure 1a). In this sense, ElSohly [17] isolated and characterized four hexahydrocannabinols from high-potency *Cannabis sativa* L., namely (6a*R*,9*S*,10a*R*)-6,6,9-trimethyl-3-pentyl-6a,7,8,9,10,10a-hexahydro-6*H*-benzo[c]chromene-1,9-diol (**2**), (6a*R*,9*R*,10a*R*)-1,9-dihydroxy-6,6,9-trimethyl-3-pentyl-8,9,10,10a-tetrahydro-6*H*-benzo[c]chromen-7(6a*H*)-one (**3**), (6a*R*,9*S*,10*S*,10a*R*)-6,6,9-trimethyl-3-pentyl-6a,7,8,9,10,10a-hexahydro-6*H*-benzo[c]chromene-1,9,10-triol (**4**), (6a*R*,9*R*,10*S*,10a*R*)-6,6,9-trimethyl-3-pentyl-6a,7,8,9,10,10a-hexahydro-6*H*-benzo[c]chromene-1,10-diol (**5**), and (6a*R*,9*S*,10a*S*)-6,6,9-trimethyl-3-pentyl-6a,7,8,9,10,10a-hexahydro-6*H*-benzo[c]chromene-1,10a-diol (**6**) (Figure 1b).

### 2.1. Synthesis of Hexahydrocannabinol and Its Analogs

**Scheme 1:**
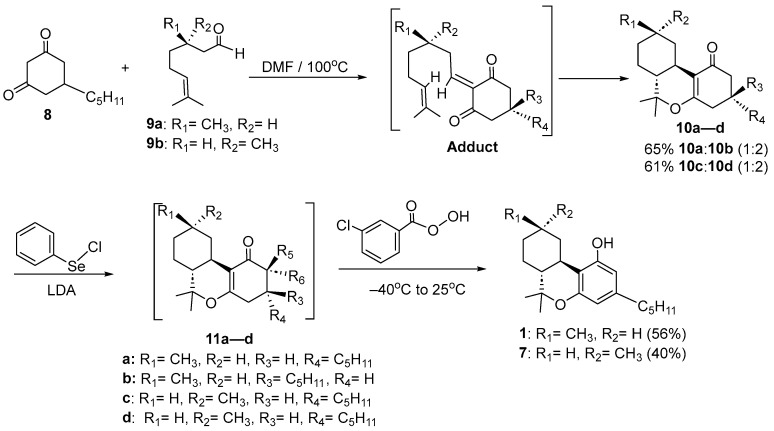
Total synthesis of (9*R*) hexahydrocannabinol (**1**) and (9*S*) hexahydrocannabinol (**6**) developed by Tietze [18].

HHC and its analogs have been achieved in two different approaches: total synthesis or partial synthesis via hydrogenation of cannabidiol analogs. The first total stereoselective synthesis of natural (6a*R*,9*R*,10a*R*)-6,6,9-trimethyl-3-pentyl-6a,7,8,9,10,10a-hexahydro-6*H*-benzo[c]chromen-1-ol (**1**) and its unnatural 6a*R*,9*S*,10a*R*)-6,6,9-trimethyl-3-pentyl-6a,7,8,9,10,10a-hexahydro-6*H*-benzo[c]chromen-1-ol (**7**) diastereomer was developed by Tietze [18] starting with 5-pentylcyclohexane-1,3-dione (**8**) and optically pure citronellal (**9a** or **9b**) via a intramolecular Diels–Alder reaction and aldol condensation followed by aromatization and elimination along a two-step reaction (Scheme 1).

The condensation between **8** and **9** generates the adduct 3,7-dimethyloct-6-en-1-ylidene)-5-pentyllcyclohexane-1,3-dione, which upon intramolecular cycloaddition, affords the substituted 1*H*-benzochromen core (**10a**–**d**). The chiral center of citronellal (*R*- or *S*-epimers) makes the cycloaddition reaction stereo-controlled. The two epimers are obtained due to the low stereoselectivity of the aldol condensation. However, this does not affect the synthesis of hexahydrocannabinol **1** and **7** since compounds **10a** and **10b** lose chirality in the subsequent aromatization step.

The aromatization step was carried out using lithium *N*,*N* diisopropylamide (LDA) to deprotonate the mixture **10a**/**10b** or **10c**/**10d** and benzeneselenenyl chloride to afford compounds **11a**/**11b** or **11c**/**11d**. 3-chlorobenzoperoxoic acid was used for the oxidation reaction to obtain compounds **1** and **7** with a 56% and 40% yield, respectively, from the last two steps. Aromatization and oxidation reactions were achieved in a one-pot reaction [19,20,21,22] without isolation of the selenide compounds **11a**–**d**.

**Scheme 2:**
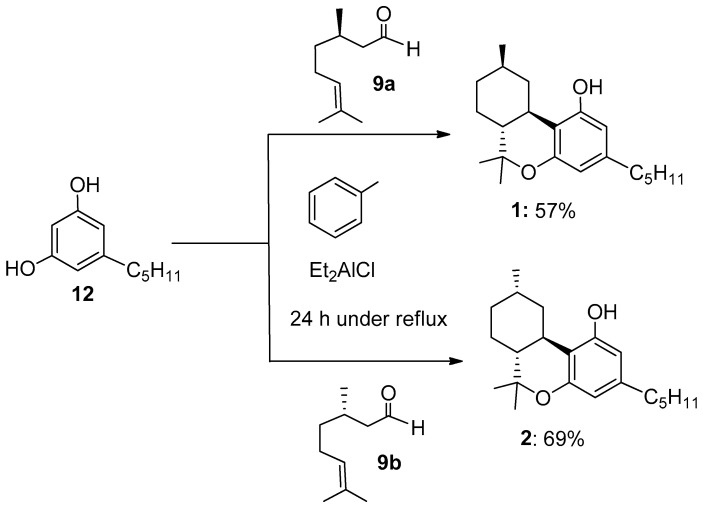
Total synthesis of (9*R*) hexahydrocannabinol (**1**) and (9*S*) hexahydrocannabinol (**7**) developed by Cornia [23].

Another methodology to synthesize (*R*)-HHC (**1**) and (*S*)-HHC (**7**) was reported by Cornia [23] using diethylaluminium chloride (Et_2_AlCl) to mediate the Knoevenagel condensation of olivetol (**12**) with (*R*)-(+)- or (*S*)-(−)-citronellal (**9a**, **9b**) followed by the intramolecular hetero Diels–Alder reaction (Scheme 2). The reaction was performed with different amounts of Et_2_AlCl and the best result was obtained with a 0.5 equivalent of Et_2_AlCl refluxing in toluene to produce **1** and **7** in a 57% and 69% isolated yield, respectively, after flash chromatography.

**Figure 2:**
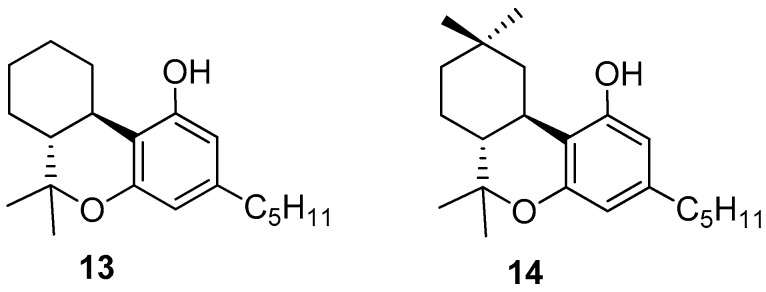
HHC homologs synthesized by Anderson [24].

Using this procedure, Anderson et al. [24] synthesized HHC homologs such as one lacking the C-11 methyl group (6a*R*,10a*R*)-6,6-dimethyl-3-pentyl-6a,7,8,9,10,10a-hexahydro-6*H*-benzo[c]chromen-1-ol (**13**) and the C-9 geminal dimethyl analog of HHC (6a*R*,10a*R*)-6,6,9,9-tetramethyl-3-pentyl-6a,7,8,9,10,10a-hexahydro-6*H*-benzo[c]chromen-1-ol (**14**) with 52% and 71% of yield, respectively (Figure 2). They reported an action mechanism for the non-electrophilic tetrahydrocannabinol derivatives (**13** and **14**) through the production of spinal antinociception mediated by TRPA1, which demonstrates that the stimulation of this ion channel could be a new approach to relieve pain.

Lee [25] employed the same hetero Diels–Alder approach, for the synthesis of (*R*) HHC (**1**) and (S) HHC (**7**), but he used ethylenediamine diacetate (EDDA) (20 mol %) as a catalyst in the presence of triethylamine (TEA) instead of Et_2_AlCl. The reaction mixture was refluxed in xylene for 24 h to afford (9*R*)-HHC (**1**) and (9*S*)-HHC (**7**) with a 72% and 73% yield, respectively.

Lee [25] extended the method to synthesize a wide group of hexahydrocannabinol derivatives using several types of resorcinols and naphthols. As seen in Table 1, the cycloaddition reactions were accomplished with resorcinols, including ester groups on the benzene ring and with 1- and 2-naphthol.

**Scheme 3:**
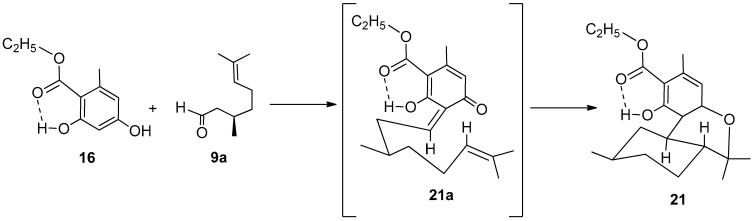
Reaction mechanism for the condensation between resorcinol derivative **15** and aldehyde **9a** followed by the intramolecular Diels–Alder reaction.

Compounds **21**, **22**, and **26** were obtained with higher yields than **20** and **25** for the presence of a carbonyl group in the ortho-position related to one of the hydroxyl groups on the phenyl ring. This fact can be elucidated due to the hydrogen bond between the hydroxyl group and the carbonyl group of the ethyl ester conferring a higher regiospecificity to the cyclization reaction, which is likely to occur at the position without hydrogen bonding. On the other hand, the stereospecificity during the intramolecular Diels–Alder reaction could be explained considering that in the transition state (**21a**), the methyl group adopted a coplanar structure in the chair configuration, so the exo-transition state is energetically more favorable than the endo-transition state, as shown in Scheme 3.

**Scheme 4:**
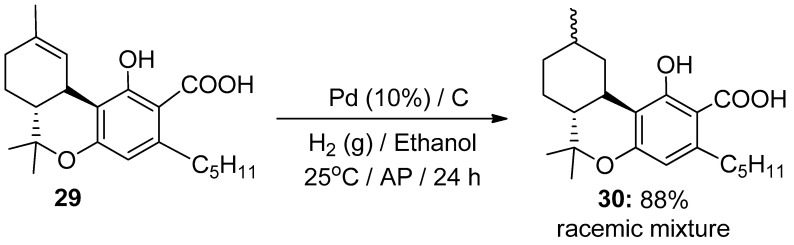
Hydrogenation reaction of HHCA-**27**.

The most common partial synthesis of HHC methodology is through the hydrogenation reaction of Δ9THC or its isomers Δ8THC and Δ10THC. Scialdone [26] reported the hydrogenation of cannabis oil produced with extraction of Cannabis sativa. The cannabis extract enriched with (6a*R*,10a*S*)-1-hydroxy-6,6,9-trimethyl-3-pentyl-6a,7,8,10a-tetrahydro-6*H*-benzo[c]chromene-2-carboxylic acid (THCA-**29**) was dissolved in absolute ethanol and treated with 10% Pd/C and hydrogen gas at room temperature and atmospheric pressure (AP), stirring overnight. The racemic mixture of diastereomers (6a*R*,10a*S*)-1-hydroxy-6,6,9-trimethyl-3-pentyl-6a,7,8,9,10,10a-hexahydro-6*H*-benzo[c]chromene-2-carboxylic acid (**30**) was obtained with 88% (Scheme 4).

**Scheme 5:**
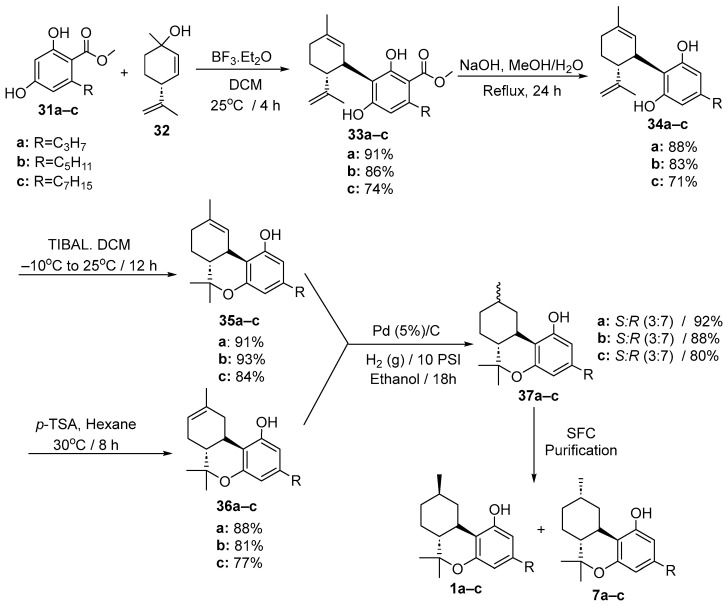
Synthetic pathway using lewis acid, bases, organometallic reagents, and hydrogenation to obtain 9*R*-HHC-**1a**–**c** and 9*S*-HHC-**6a**–**c**.

Another example for the synthesis of HHC derivatives was developed by Cruces et al. [10,27] starting with carboxymethyl ester of olivetol analogs. As shown in Scheme 5, methyl 2,4-dihydroxy-6-alkylbenzoate analogs (**31a**–**c**) were coupled with (4*R*)-1-methyl-4-(prop-1-en-2-yl)cyclohex-2-enol (**32**) using boron trifluoride–etherate as a catalyst and dichloromethane as a solvent to obtain the (1′*S*,2′*R*) -methyl 2,6-dihydroxy-5′-methyl-4-alkyl-2′-(prop-1-en-2-yl)-1′,2′,3′,4′-tetrahydro-[1,1′-biphenyl]-3-carboxylate derivatives (**33a**–**c**). It was followed by the hydrolysis reaction with sodium hydroxide in methanol:H_2_O to afford (1′*S*,2′*R*)-5′-methyl-4-alkyl-2′-(prop-1-en-2-yl)-1′,2′,3′,4′-tetrahydro-[1,1′-biphenyl]-2,6-diol analogs (**34a**–**c**). The cyclization reaction was carried out using triisobutylaluminum (TIBAL) as Lewis’s acid catalyst to attain Δ9-THC- **35a**–**c** or using *p*-toluene sulfonic acid (*p*-TSA) as a protic acid catalyst to afford Δ8-THC- **36a**–**c**. Δ9-THC and Δ8-THC were hydrogenated using 5% Pd/C in ethanol to yield 9*S* and 9*R*-(6a*R*,10a*S*)-6,6,9-trimethyl-3-alkyl-6a,7,8,9,10,10a-hexahydro-6*H*-benzo[c]chromen-1-ol diastereomers in a ratio of 3:7 (**37a**–**c**) with 80–92% of yield. The pure diastereomers, (6a*R*,9*R*,10a*S*)-6,6,9-trimethyl-3-alkyl-6a,7,8,9,10,10a-hexahydro-6*H*-benzo[c]chromen-1-ol (**1a**–**c**) and (6a*R*,9*S*,10a*S*)-6,6,9-trimethyl-3-alkyl-6a,7,8,9,10,10a-hexahydro-6*H*-benzo[c]chromen-1-ol (**7a**–**c**), were separated with supercritical fluid chromatography (SFC) using a chiral column [10]. It has been observed that the catalytic hydrogenation of Δ9-THC using Adam’s catalyst affords the (9*S*)-HHC and (9*R*)-HHC isomers in approximately a 1:7 ratio [28]. Moreover, Venkateswara [29] demonstrated that hydrogenation of a cyclohex-3-enone core in the presence of H_2_–Pd/C (10 mol %) afforded *S*- and *R*-diastereomers in a 3:6 ratio, whereas under H_2_–PtO_2_ (Adam’s catalyst) conditions, *R*- and *S*-diastereomers were obtained in a 2:8 ratio.

Garg and coworkers [30] established a method to obtain the (9*R*-)HHC diastereomer as a major product via the hydrogen-atom transfer reduction of D8THC, avoiding potentially risky catalytic hydrogenation conditions and poisonous heavy metals such as platinum or palladium. (9*R*-)HHC has been evaluated for the treatment of colon cancer [31] and ocular hypotony [32] with promising results. Furthermore, separately investigating the biological properties of the 9*R*-HHC (**1**) diastereomer would offer a comprehension of its pharmaceutical activity.

**Scheme 6:**
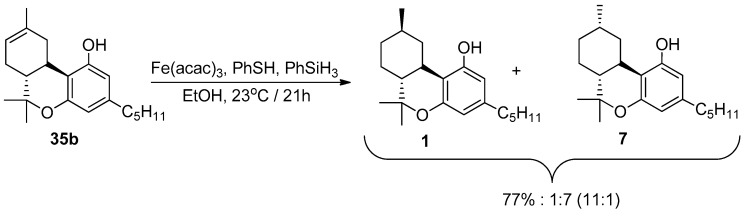
Synthesis of HHC diastereomers (**1** and **7**) via hydrogen-atom transfer reduction of D8THC (**35b**).

They employed tris(acetylacetonato)iron(III) that is an effective hydrogen atom donor catalyst for the radical reduction reactions in combination with thiophenol and silylbenzene to reduce d8THC (**35b**). Under these conditions, the mixture of diastereomers afforded 77% of yield and a ratio of 11:1 (9*R*-HHC:9*S*-HHC) (Scheme 6). It is outstanding that the hydrogen-atom transfer conditions furnish the highest diastereoselectivity in favor of the equatorial orientation of the methyl group at position 9, demonstrating that 9*R*-HHC is energetically favorable.

### 2.2. Pathways to Obtain Natural Machaeriols and Their Synthetic Analogs

**Figure 3:**
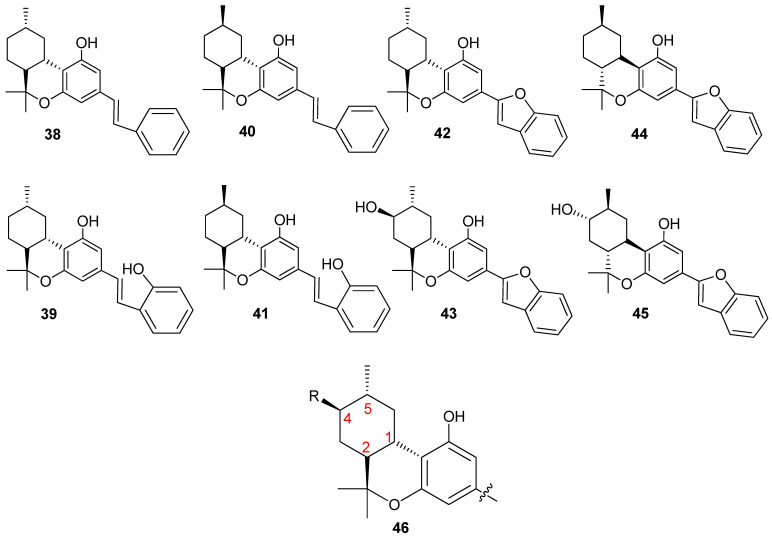
Structures of the natural (+)-machaeriol A–D (**38**, **39**, **42**, and **43**), unnatural (−)-machaeriol A−D (**40**, **41**, **44**, and **45**), and hexahydrodibenzopyran (HHDBP) scaffold (**46**). Red number indicated the stereochemistry configuration.

A novel class of HHC analogs, machaeriols, were isolated from the stem bark of *Machaerium multiflorum* at the beginning of the 21st century [33,34,35] such as (6a*R*,9*S*,10a*S*)-6,6,9-trimethyl-3-((*E*)-styryl)-6a,7,8,9,10,10a-hexahydro-6*H*-benzo[c]chromen-1-ol (**38**), (6a*R*,9*S*,10a*S*)-3-((*E*)-2-hydroxystyryl)-6,6,9-trimethyl-6a,7,8,9,10,10a-hexahydro-6*H*-benzo[c]chromen-1-ol (**39**), (6a*R*,9*S*,10a*S*)-3-(benzofuran-2-yl)-6,6,9-trimethyl-6a,7,8,9,10,10a-hexahydro-6*H*-benzo[c]chromen-1-ol (**42**), and (6a*R*,8*R*,9*R*,10a*S*)-3-(benzofuran-2-yl)-6,6,9-trimethyl-6a,7,8,9,10,10a-hexahydro-6*H*-benzo[c]chromene-1,8-diol (**43**) (Figure 3). However, there are few reports related to the total synthesis of these hydrogenated cannabinoids because the stereo-controlled construction of the stereocenters of the hexahydrodibenzopyran (**46**) ring depicts a notable synthetic challenge. Elsoy [34] and Muhammad [35] evaluated the activity of compounds **38** and **42** as antimalarial antileishmanial agents and compound **42** exhibited an IC_50_ of 120 nM against a *Plasmodium falciparum* W-2 clone and 900 nM against *Leishmania donavani*. Also, compound **38** presented antibacterial action against *S. aureus* and MRSA with an IC_50_ of 2.6 μM and antifungal activity against *Candida albicans* (IC_50_, 3.5 μM). The resemblance in the scaffold of all these compounds with D9THC and HHC motivated some scientists to develop different pathways to obtain them.

The first total synthesis of natural (+)-machaeriol D (**43**) was developed by Pan [36].

**Scheme 7:**
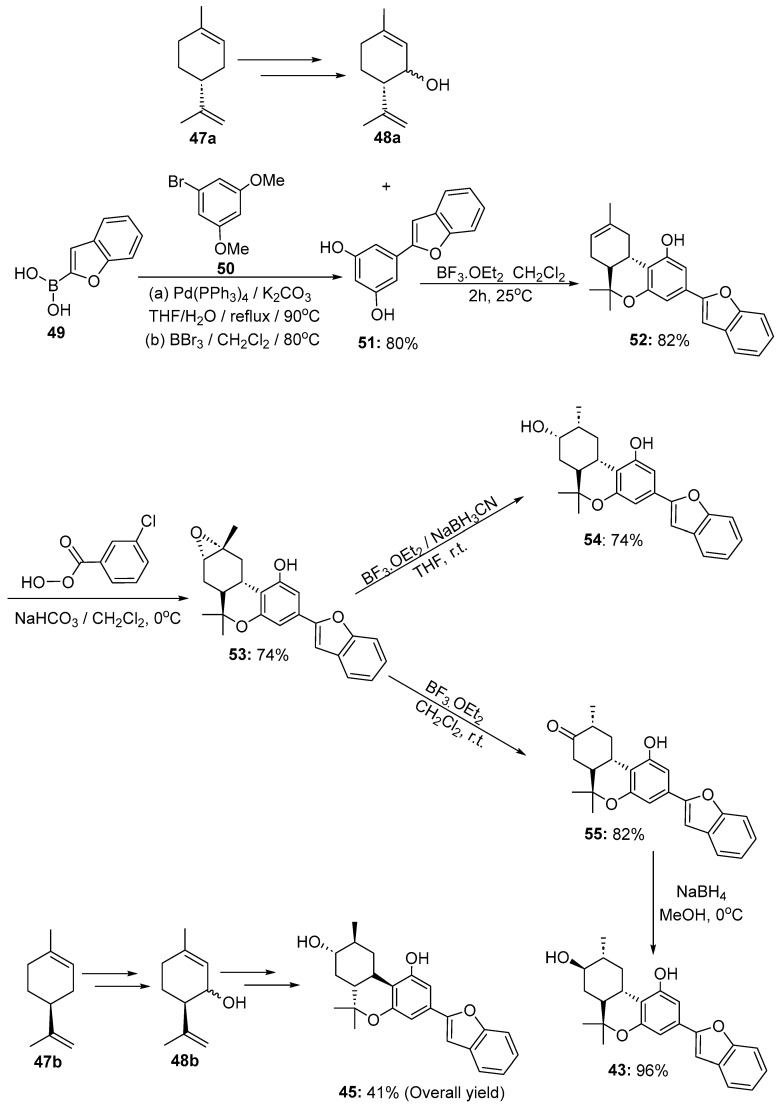
Pathway to synthesize (+) and (−)-machaeriol-D-(**43**/**45**) and epimachaeriol-D (**54**).

The key point in the synthetic route was a highly regio- and sereoselective S_N_2′ reaction to afford the 5-methyl-2-((prop-1-en-2-yl)cyclohexyl)benzene-1,3-diol scaffold (**46**) with the four stereocenters (C1, C2, C4, and C5) present in the final molecule (Figure 3). The main disadvantage of this method is that 18 synthesis steps are required, entailing that the overall yield of (+)-machaeriol D is lower than 10%. Dethe [37] improved this procedure by applying an atom economical and protecting group-free synthetic strategy with less than six operational steps starting with *R*-(+) and *S*-(−)-limonene (**47**). This pathway provides the synthesis of both natural product **43** and its enantiomer **45** (Scheme 7).

The first step consists of the diastereoselective-coupling reaction between allylic alcohol **45** obtained from *S*-(−)- limonene (**47**) with benzofuran-benzene-diol (**51**) in the presence of BF_3_·OEt_2_ followed by isomerization of the double bond to generate a 90% isolate yield of *trans*- hexahydrodibenzopyran compound **52**. The high diastereoselectivity showed is due to the bulky isopropenyl group in the allyl alcohol. The second step involved the Prilezhaev epoxidation, which was carried out using 3-chlorobenzoperoxoic acid (*m*-CPBA) to afford a 74% yield of epoxide **53**. The reaction was highly stereospecific, and it occurred from the *α*-face to obtain only one diastereoisomer. Interestingly, the regioselective opening of epoxide **53** occurs in the presence of the mixture of sodium cyanoborohydride (NaBH_3_CN) and BF_3_·OEt_2_ (1:1) to obtain the epimer of machaeriol-D (**54**) with a 45% overall yield. On the other hand, epoxide **53** undergoes a semipinacol rearrangement catalyzed by BF_3_·OEt_2_ to produce, regioselectively, ketone **55** with 82% of yield. The last step represents the reduction of **55** using sodium borohydride to afford the natural product (+)-machaeriol-D (**43**) in a 96% yield and 48% of overall yield.

In a similar fashion, the unnatural (-)-machaeriol-D-**45** was synthesized starting from *R*-(+)-limonene (**48b**) (Scheme 6) with a 41% overall yield.

**Scheme 8:**
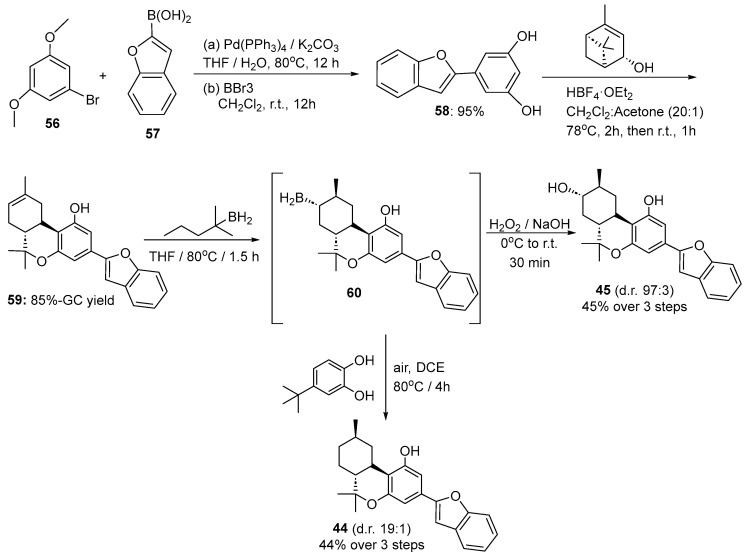
Synthesis of (−)-machaeriol B (**44**) and (−)-machaeriol D (**45**) developed by Studer [38] using lewis acids, palladium catalysts, boronic reagents, and base/peroxides.

Moreover, Studer [38] reported the synthesis of (−)-machaeriol B (**44**) and (−)-machaeriol D (**45**) focusing on the Friedel–Crafts alkylation of 5-(benzofuran-2-yl)benzene-1,3-diol (**58**), which was obtained in a 95% yield via the Suzuki–Miyaura cross-coupling reaction between **56** and **57**, with (*S*)-cis-verbenol, followed by the cyclization that enables the building of the tetrahydrodibenzopyran motif of Δ8THC. (6a*R*,10a*S*)-3-(benzofuran-2-yl)-6,6,9-trimethyl-6a,7,8,10a-tetrahydro-6*H*-benzo[c]chromen-1-ol (**59**) was accomplished with 85% of yield. The next step was the hydroboration of the double bond in **59**. To achieve a high diastereoselective reaction, they used thexylborane followed by oxidation with sodium hydroxide and peroxide to afford (6a*R*,8*S*,9S,10a*S*)-3-(benzofuran-2-yl)-6,6,9-trimethyl-6a,7,8,9,10,10a-hexahydro-6*H*-benzo[c] chromene-1,8-diol (**45**) in a 45% overall yield for the last three steps (cyclization/hydroboration/oxidation) with 97:3 selectivity (measured using GC-FID) (Scheme 8).

Also, they synthesized (6a*R*,9*R*,10a*S*)-3-(benzofuran-2-yl)-6,6,9-trimethyl-6a,7,8,9,10,10a-hexahydro-6*H*-benzo[c]chromen-1-ol (**44**) starting with hydroboration intermediate **60** applying the hydrodeborylation radical chain reaction. In this sense, they used the procedure developed by Renaud [38] for the conversion of organoborons to the appropriate alkanes under an air atmosphere with the addition of 4-*tert*-butylcatechol (Scheme 8). Compound **44** was isolated in a 44% yield over three steps (cyclization/hydroboration/protodeborylation) with 19:1 selectivity.

Summarizing, Studer accomplished the synthesis of (−)-machaeriol B (**45**) and D (**44**) in 43% and 42% overall yields over five steps using a Friedel–Crafts coupling reaction and highly diastereoselective hydroboration [39] followed by either an oxidative or reductive way. This route represents the best yield in the fewest steps without protecting groups reported so far for the synthesis of unnatural machaeriol B and D.

**Scheme 9:**
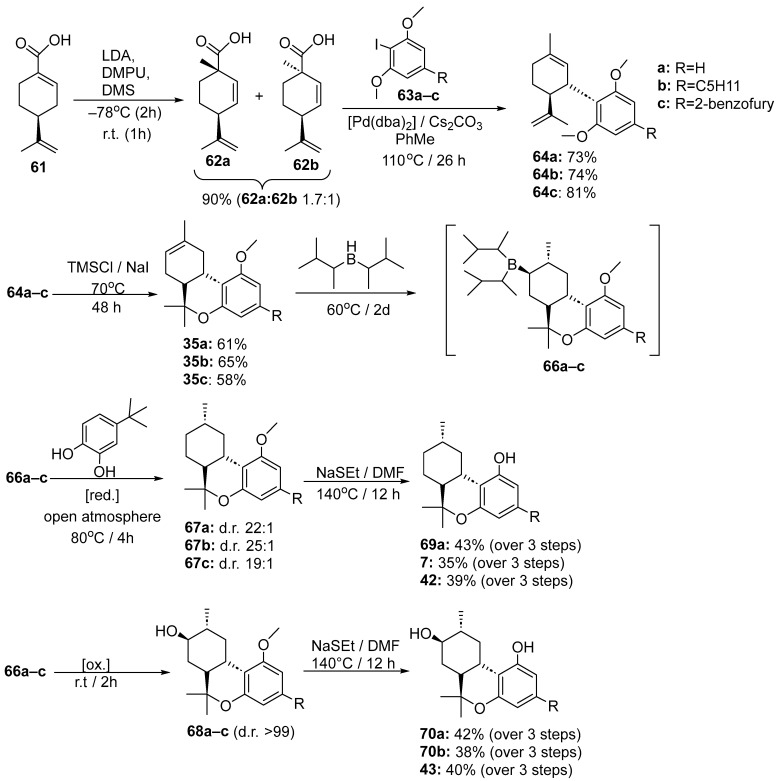
Synthesis of (6a*R*,9*S*,10a*S*)-6,6,9-trimethyl-6a,7,8,9,10,10a-hexahydro-6*H*-benzo[c]chromen-1-ol (**69a**), (6a*R*,8*R*,9*R*,10a*S*)-6,6,9-trimethyl-6a,7,8,9,10,10a-hexahydro-6*H*-benzo[c]chromene-1,8-diol (**70a**), (6a*R*,9*S*,10a*S*)-6,6,9-trimethyl-3-pentyl-6a,7,8,9,10,10a-hexahydro-6*H*-benzo[c]chromen-1-ol (**7**), (6a*R*,8*R*,9*R*,10a*S*)-6,6,9-trimethyl-3-pentyl-6a,7,8,9,10,10a-hexahydro-6*H*-benzo[c]chromene-1,8-diol (**70b**), (6a*R*,9*S*,10a*S*)-3-(benzofuran-2-yl)-6,6,9-trimethyl-6a,7,8,9,10,10a-hexahydro-6*H*-benzo[c]chromen-1-ol (**42**), and (6a*R*,8*R*,9*R*,10a*S*)-3-(benzofuran-2-yl)-6,6,9-trimethyl-6a,7,8,9,10,10a-hexahydro-6*H*-benzo[c]chromene-1,8-diol (**43**) developed by Studer [40].

Later, Studer [40] established a five-step route to obtain *S*-HHC (**7**), natural machaeriol B (**42**), D (**43**), and their analogs (6a*R*,9*S*,10a*S*)-6,6,9-trimethyl-6a,7,8,9,10,10a-hexahydro-6*H*-benzo [*c*]chromen-1-ol (**69a**) and (6a*R*,8*R*,9*R*,10a*S*)-6,6,9-trimethyl-6a,7,8,9,10,10a-hexahydro-6*H*-benzo[c]chromene-1,8-diol (**70a**) as Scheme 9 shows. They began their synthetic approach with the regioselectivity alkylation of commercially available (*S*)-4-(prop-1-en-2-yl)cyclohex-1-enecarboxylic acid (**61**) to obtain (1*S*,4*S*)- 1-methyl-4-(prop-1-en-2-yl)cyclohex-2-enecarboxylic acid **62a** and (1*R*,4*S*)-1-methyl-4-(prop-1-en-2-yl)cyclohex-2-enecarboxylic acid (**62b**) with 90% of yield and 1.7:1 diastereoselectivity. To control the *α*/*γ* regioselectivity, they used lithium *N*,*N*diisopropyl amide (LDA) in a THF/DMPU mixture to generate the dienolate from **61,** which reacted with dimethyl sulfate (DMS) to yield carboxylic acid **62a**,**b** with complete *α*-selectivity. After that, a stereospecific decarboxylative g-arylation was carried out over the mixture of diastereomers (**62a**,**b**) using bis(dibenzylideneacetone)palladium, cesium carbonate, and 2-iodo-1,3-dimethoxybenzene derivatives (**63a**–**c**) to generate (1*S*,2*S*)-2′,6′-dimethoxy-5-methyl-2-(prop-1-en-2-yl)-1,2,3,4-tetrahydro-1,1′-biphenyl derivatives (**64a**–**c**) in a 73, 74, and 81% yield, respectively, as single diastereomers. They demonstrated that diastereomer **62b** does not undergo *γ*-arylation (Scheme 9).

The next step is the formation of Δ8-tetrahydrodibenzopyran derivatives (**65a**–**c**) through the selective deprotection of one methyl ether followed by cyclization and isomerization in a one-pot reaction using trimethylsilyl chloride (TMSCl) and sodium iodide (NaI). The heterogeneous hydrogenation of **65a**–**c** compounds in the presence of Pt_2_O/C affords the mixture of 3:1 *R*-:*S*-diastereomers. To succeed in this stereoselective issue, they explored the hydroboration using disiamylborane (Sia_2_BH) and succeeding radical reduction with 4-*tert*-butylcatechol to furnish (6a*R*,9*S*,10a*S*)-1-methoxy-6,6,9-trimethyl-6a,7,8,9,10,10a-hexahydro-6*H*-benzo[c]chromene derivatives **67a**–**c** in acceptable yields and excellent diastereoselectivities (17:1 for **67a**, 19:1 for **67b**, and 22:1 for **67c**). In addition, the hydroboration of **65a**–**c** using Sia_2_BH followed by the oxidative reaction in the presence of H_2_O_2_ and NaOH provided (6a*R*,8*R*,9R,10a*S*)-1-methoxy-6,6,9-trimethyl-6a,7,8,9,10,10a-hexahydro-6*H*-benzo[c]chromen-8-ol (**68a**–**c**) as single diastereoisomers (d.r. > 99:1) in good yields. The removal of methyl groups, as the last step, was easily attained with ethanethiol sodium salt (NaSEt) in DMF under reflux to obtain the desired products. Therefore, *S*-HHC (**7**), (+)-machaeriol B (**42**), and (+)-machaeriol D (**43**) were synthesized in just five steps in a 22%, 18%, and 19% overall yield, respectively.

### 2.3. Partial and Total Synthesis of 9R-11-Hydroxyhexahydrocannabinol and Its Derivatives

(6a*S*,10a*R*)-9-(hydroxymethyl)-6,6-dimethyl-3-pentyl-6a,7,8,9,10,10a-hexahydro-6*H*-benzo[c]chromen-1-ol (9*R*-11-hydroxyhexahydrocannabinol-**71**) was isolated as one of the minor metabolites of Δ9-THC after treating mice (male, Charles River CDl, 23 g) with Δ9-THC (100 mg/kg, i.p.) suspended in Tween 80 and isotonic saline administered at 26 h and 2 h before death by stunning and decapitation [41]. Also, it was the major metabolite formed using the incubation of 9*R*-HHC with hepatic microsomes of rats, guinea pigs, and rabbits [42]. Interestingly, this compound was established to be closely seventeen times more active than Δ9 THC; for these reasons, great interest arose to develop the synthesis of it to deeply study its pharmacological activity in vitro and in vivo as well as its toxicity [43].

**Scheme 10:**
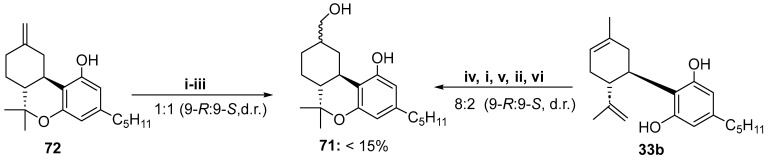
Partial synthetic approach of **71** reported by Skinner [33] and Kozela [44]. **i**: Acetic anhydride, pyridine, r.t. 2h; **ii**: BH_3_, THF, 0 °C, 1 h, then Me_3_N^+^O^−^·2H_2_O, reflux 2 h; **iii**: NaOH(1M), MeOH, 2 h; **iv**: *p*-TSA, hexane, 72h; **v**: SeO_2_, ethanol, 24 h; **vi**: Pd/C, H_2_ (1 atm), 24 h methanol.

There are some reports that have described the partial synthesis of 11-hydroxyhexahydrocannabinol as a mixture of diastereomers. The first one was developed by Skinner [33] starting from (6a*S*,10a*R*)-6,6-dimethyl-9-methylene-3-pentyl-6a,7,8,9,10,10a-hexahydro-6H-benzo[c]chromen-1-ol (**72**) in three steps with 1:1 (dr, 9*R*:9*S*). The second one was established by Kozela [44] beginning with CBD (**33b**) in five steps with 8:2 (dr, 9*R*:9*S*) (Scheme 10). The overall yields in both synthetic routes are lower than 15%.

The first total synthesis of compound **71** was reported by Appayee [45] applying the inverse-electron-demand Diels–Alder reaction to afford, stereoselectively, six-membered ring compound **75** starting with 6-((tert-butyldimethylsilyl)oxy)hexa-2,4-dienal (**73**) and 2-(2,6-dimethoxy-4-pentylphenyl)acetaldehyde (**74**) and catalyzed by (*S*)-pyrrolidine-2-carboxylic acid. (1*R*,6*R*)-6-(((tert-butyldimethylsilyl)oxy)methyl)-2′,6′-dimethoxy-4′-pentyl-1,6-dihydro-[1,1′-biphenyl]-3-carbaldehyde (**75**) was obtained with a 72% yield and 97% enantioselectivity.

**Scheme 11:**
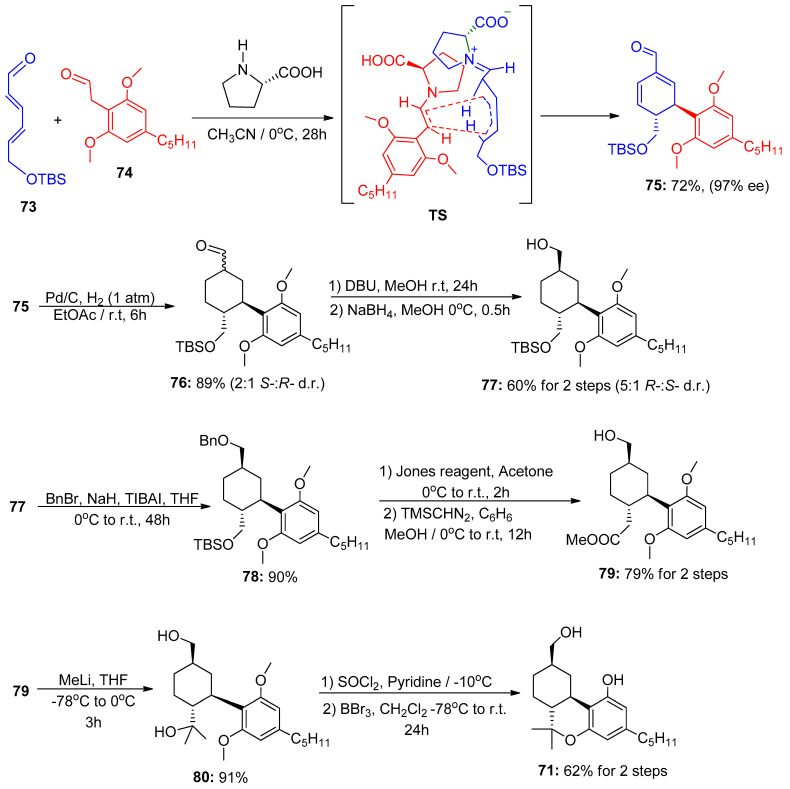
Total synthesis of 9*R*-11-hydroxyhexahydrocannabinol developed by Appayee [45].

In the second step, 3-carbaldehyde (**75**) was treated with hydrogen under Pd/C to yield saturated carbaldehyde (**76**) as a racemic mixture (Scheme 11). Appayee [45] used DBU in MeOH followed by the in situ reduction of the epimerized product to achieve a good diastereoselectivity (5:1, d.r.) of cyclohexyl methanol (**77**) with a 60% yield after two steps. The conversion of **77** to t 2-((1*R*,2*R*,4*R*)-2-(2,6-dimethoxy-4-pentylphenyl)-4-(hydroxymethyl) cyclohexyl)propan-2-ol (**80**) was accomplished in four steps starting with the benzyl protection of the carbinol, then the direct oxidation of the silyl ethers’ ether using the Jones reagent followed by treatment with trimethylsilyidiazomethanhexane resulting in cyclohexyl acetate (**79**) 16. Finally, the addition of methyllithium to compound **79** afforded cyclohexylpropan-2-ol (**80**) with an 85% yield after four steps (Scheme 11).

**Scheme 12:**
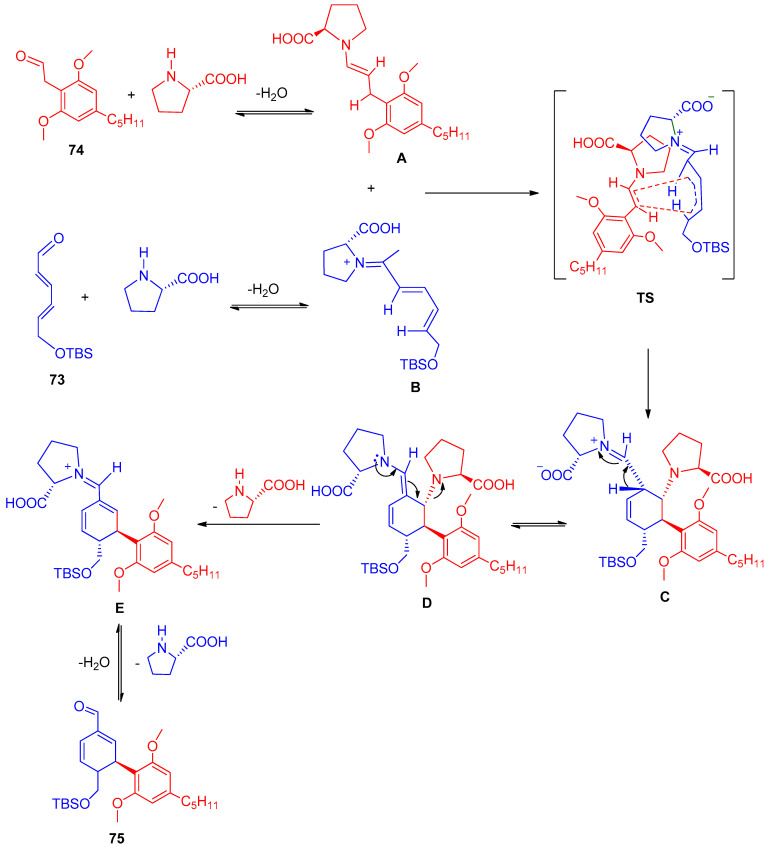
Mechanism for the IEDDA reaction proposed by Appayee [46].

In this inverse-electron-demand Diels–Alder (IEDDA) reaction, an electron-rich dienophile (**74**) undergoes a 1,4 addition with an electron-poor diene (**73**). A tentative mechanism for this IEDDA was proposed by Appayee [46]. One equivalent of (*S*)-pyrrolidine-2-carboxylic acid reacts with diene **73** to afford an enamine intermediary A, and the second equivalent of (*S*)-pyrrolidine-2-carboxylic acid reacts with dienophile (**74**) to furnish an iminium intermediary B. A and B go through a possible transition state, TS, to generate adduct C. The enolization of C leads to the formation of an enamine intermediary D. The last two steps of the mechanism comprise the elimination of the catalyst to give iminium intermediate E and the hydrolysis of E to furnish compound **75** (Scheme 12).

The last step was a challenge due to the presence of a free tertiary alcohol group in **80** that triggers multiple dehydrated and rearranged products during the deprotection and cyclization reactions. For this reason, Appayee [45] decided to transform compound **80** into a terminal alkene and treated it with boron tribromide to obtain 9*R*-11-hydroxyhexahydrocannabinol (**71**) with a 24% overall yield (Scheme 11).

### 2.4. C-9 Ketocannabinoids: Different Enantioselective Synthetic Routes

**Scheme 13:**
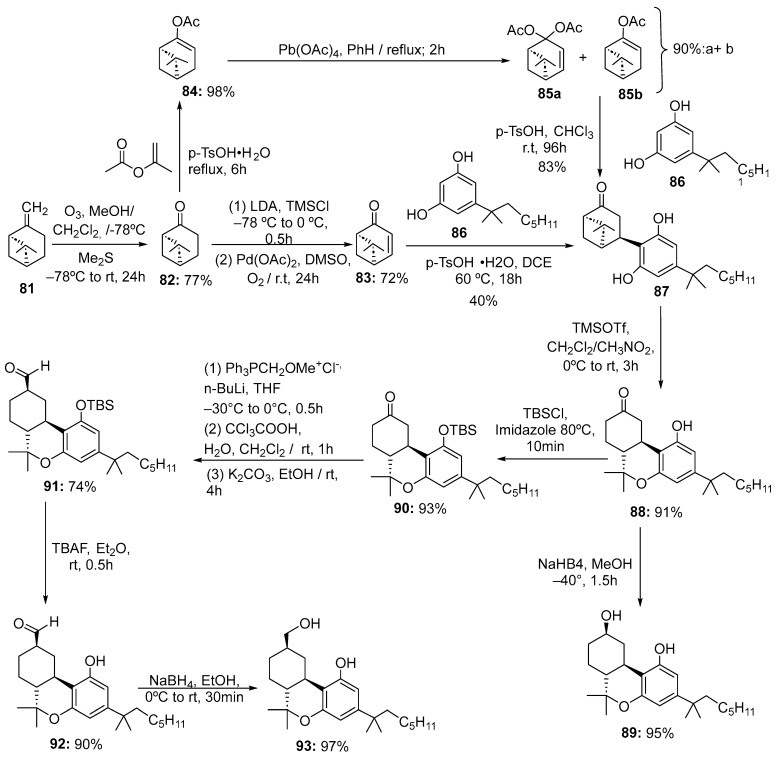
Total synthesis of (−)-nabilone (**88**), canbisol (**89**), 9*R*-aldehyde nabilone derivative (**92**), and 9*R*-hydroxymethyl nabilone derivative (**93**).

The first synthesis of a C9-ketocannabinoid related to enantioenriched nabilone (**88**) was first developed by Archer and coworkers at Eli Lilly Company in 1977 [47]. Nabilone’s structure is comparable to that of THC. Both compounds are a dibenzopyran core, with a dimethyl at C6, and a hydroxyl at C1. Contrasting THC, nabilone has a dimethylheptyl lipophilic chain at C3, a saturated ring at the terpene scaffold, and a ketone group instead of a methyl group in C9. Pertwee [48] demonstrated that due to these structural differences, nabilone is more potent than THC, producing higher cAMP agonist and [^35^S]GTP*γ*S binding affinity in mouse brain tissue. In 1975, Lemberger and Rowe [49] reported the use of nabilone administration in humans, pointing out that the behavioral effects begin at about 1 h after administration, and last for 8–9 h. In total, 5 mg of nabilone produced dry mouth, euphoria, tachycardia, and postural hypotension. These effects were insignificant at 2.5 mg, and lacking at 1 mg. Later, clinical studies advocated that nabilone may be effective in relieving agitation in patients with dementia [50,51], nightmares in patients with post-traumatic stress disorder [52], and non-motor symptoms in patients with Parkinson’s disease [53]. This motivated many research groups to improve the synthetic procedure proposed by the Eli Lilly Company and try to obtain pure diastereomers instead of the racemic mixture.

(1*R*,4*R*,5*S*)-4-(2,6-dihydroxy-4-(2-methyloctan-2-yl)phenyl)-6,6-dimethyl bicyclo[3.1.1]heptan-2-one (**88**) was produced in the four-step synthetic pathway, starting from inexpensive (1*S*,5*R*)-6,6-dimethyl-2-methylenebicyclo[3.1.1]heptane (**81**). However, the overall yield of Nabilone **88** was lower than 10%. This was assumed to be provoked by the lack of reactivity of (1*R*,5*S*)-6,6-dimethylbicyclo[3.1.1]hept-3-en-2-one (**83**). This led Nikas [54] and later Blaazer [55] and Makriyannis [56,57] to develop an alternative route of synthesis in five steps through the mixture of both terpene acetates **85a** and **85b**. The diacetates (**85**) were synthesized via the transesterification of (1*R*,5*R*)-6,6-dimethylbicyclo[3.1.1]heptan-2-one (**82**) with isopropenyl acetate to give (1*R*,5*R*,6*S*)-6-methylbicyclo[3.1.1]hept-2-en-2-yl acetate (**84**), which was then treated with lead tetraacetate in refluxing benzene. The Michael addition of resorcinol **86** to the mixture of terpene acetates **85** using *p*-toluenesulfonic acid monohydride as a catalyst and heating in DCE produced an 83% yield of Michael adduct **87,** which cyclized in the presence of trimethylsilyl triflate (TMSOTf) to obtain (6a*S*,10a*R*)-1-hydroxy-6,6-dimethyl-3-(2-methyloctan-2-yl)-7,8,10,10a-tetrahydro-6*H*-benzo[c]chromen-9(6a*H*)-one (**88**) with a 54% overall yield after five steps (Scheme 13). The reduction of **88** with sodium borohydride furnished (6a*S*,9*R*,10a*R*)-6,6-dimethyl-3-(2-methyloctan-2-yl)-6a,7,8,9,10,10a-hexahydro-6*H*-benzo[c]chromene-1,9-diol (**89**).

Makriyannis [56] reported the synthesis of (6a*S*,9*R*,10a*R*)-9-(hydroxymethyl)-6,6-dimethyl-3-(2-methyloctan-2-yl)-6a,7,8,9,10,10a-hexahydro-6*H*-benzo[c]chromen-1-ol (**93**) from **88** using the Wittig olefination of (6a*S*,10a*R*)-1-((tert-butyldimethylsilyl)oxy)-6,6-dimethyl-3-(2-methyloctan-2-yl)-7,8,10,10a-tetrahydro-6*H*-benzo[c]chromen-9(6a*H*)-one (**90**) to produce an E/Z mixture of methoxymethylene derivatives, which were hydrolyzed with trichloroacetic acid to a mixture of diastereomeric C9 aldehydes. The epimerization of this mixture afforded thermodynamically more stable 9*R*-carbaldehyde **92**. Finally, reduction with sodium borohydride in ethanol led to (6a*S*,9*R*,10a*R*)-9-(hydroxymethyl)-6,6-dimethyl-3-(2-methyloctan-2-yl)-6a,7,8,9,10,10a-hexahydro-6*H*-benzo[c]chromen-1-ol (**93**) (Scheme 13).

### 2.5. Cannabinoid Lactones Modified in the C-Ring

Makriyannis [58] substituted the C-ring in the nabilone structure with a seven-membered lactone. The goal of this work was to incorporate a labile group as lactone into the C-9 ketocannabinoid lead scaffold to improve pharmacokinetic/pharmacodynamic properties of cannabinoids and mimic their cannabinergic effects.

**Scheme 14:**
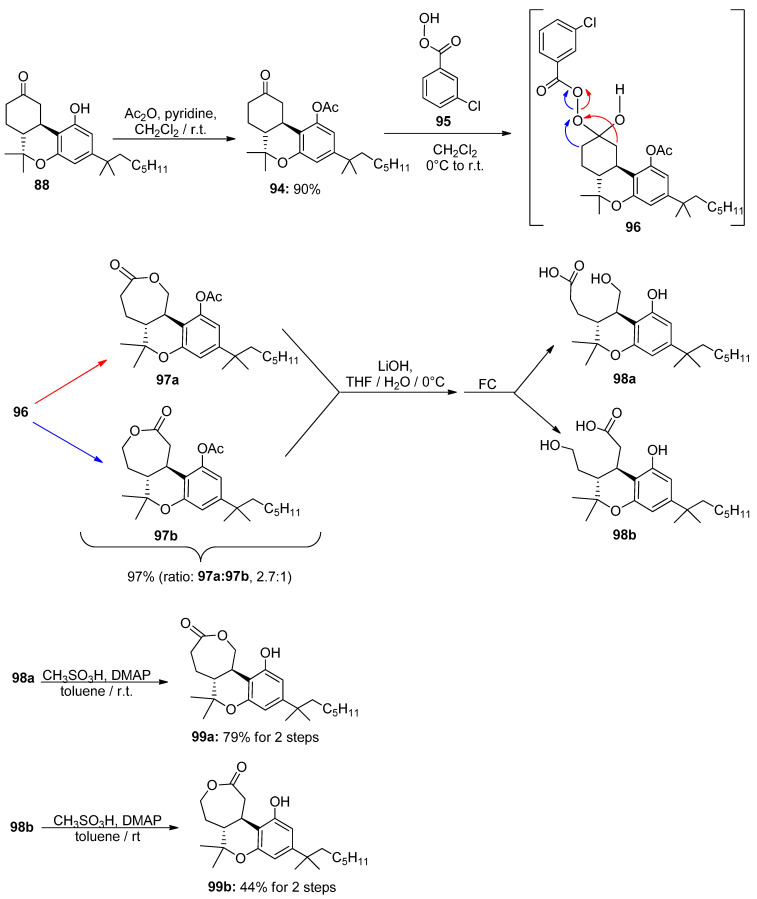
Synthesis of (5a*R*,11b*R*)-11-hydroxy-6,6-dimethyl-9-(2-methyloctan-2-yl)-4,5,5a,6-tetrahydro-1*H*-oxepino[4,3-*c*]chromen-3(11b*H*)-one (**99a**) and (5a*R*,11b*R*)-11-hydroxy-6,6-dimethyl-9-(2-methyloctan-2-yl)-4,5,5a,6-tetrahydro-1*H*-oxepino[4,5-*c*]chromen-2(11b*H*)-one (**99b**).

The synthetic pathway started with the acetylation reaction to protect the hydroxyl group in nabilone (**88**) obtaining (6a*S*,10a*R*)-6,6-dimethyl-3-(2-methyloctan-2-yl)-9-oxo-6a,7,8,9,10,10a-hexahydro-6*H*-benzo[c]chromen-1-yl acetate (**94**) with 90% of yield. It was afterward treated with 3-chlorobenzoperoxoic acid (**95**) to furnish a mixture of regioisomeric lactones **97a** and **97b** in a 97% yield after Baeyer–Villiger rearrangement via tetrahedral intermediate **96** (Scheme 14).

The regioisomers **97a** and **97b** were not able to separate, so they were reacted with lithium hydroxide to remove the acetyl group and open the lactone ring to obtain the mixture of corresponding propanoic acids **98a** and **98b** that were separated with flash column chromatography. Finally, the intramolecular cyclization was carried out in each regioisomer in the presence of methanesulfonic acid and 4-dimethylaminopyridine to generate (5a*R*,11b*R*)-11-hydroxy-6,6-dimethyl-9-(2-methyloctan-2-yl)-4,5,5a,6-tetrahydro-1*H*-oxepino[4,5-*c*]chromen-2(11b*H*)-one (**99b**) and (5a*R*,11b*R*)-11-hydroxy-6,6-dimethyl-9-(2-methyloctan-2-yl)-4,5,5a,6-tetrahydro-1*H*-oxepino[4,3-*c*]chromen-3(11b*H*)-one (**99a**) with a 44% and 79% overall yield, respectively (Scheme 14).

**Scheme 15:**
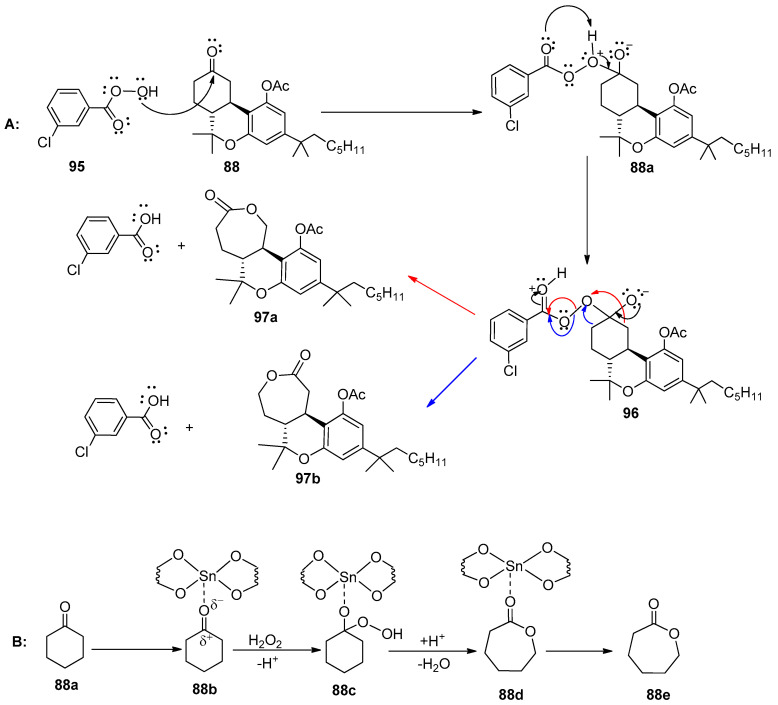
(**A**) Mechanism of Baeyer–Villiger oxidation/rearrangement in the presence of 3-chlorobenzoperoxoic acid (**95**) to generate regioisomeric cannabinergic C-ring lactones **97a** and **97b**. (**B**) Mechanism of Baeyer–Villiger oxidation/rearrangement in the presence of aqueous hydrogen peroxide as an oxidant and Sn-zeolite as a catalyst.

The Baeyer–Villiger oxidation rearrangement is a key point in this synthetic pathway; hence, we decided to include the reaction mechanism. It involves the formation of a seven-member cyclic ortho-ester from a six-member cyclic ketone using peroxyacids as an oxidant. The reaction implies the initial addition of peroxide to the carbonyl carbon to obtain an **88a** adduct, which undergoes a rearrangement to obtain the intermediate *α*−acylperoxy hemiacetals’ (Criegee) intermediary **96**. The last step compromises the alkyl migration to give the two regioisomers **97a** and **97b** with a ratio of 2.7:1 (**97a**:**97b**) (Scheme 15A). The group anti-periplanar alignment to the dissociating peroxide bond is expected to have a higher migratory ability for conformational and stereoelectronic requirements with the lower energy in the dipole interaction. Thus, the formation of lactone **97a** is favored over regioisomer **97b**. Mikami [59] investigated the regioselectivity of the Baeyer–Villiger reaction in six-membered cyclic ketones and developed a regioselective procedure to afford only one regioisomer of lactones using aqueous hydrogen peroxide as an oxidant and Sn-zeolite as a catalyst (Scheme 15B). It could be applied to the synthesis of (5a*R*,11b*R*)-11-hydroxy-6,6-dimethyl-9-(2-methyloctan-2-yl)-4,5,5a,6-tetrahydro-1*H*-oxepino[4,3-*c*]chromen-3(11b*H*)-one (**99a**).

## 3. Hydrogenated Bicyclic Cannabidiol Analogs

Cannabidiol (CBD, **34b**) is a naturally occurring compound biosynthesized within the *Cannabis sativa* plant. CBD has gained significant attention in recent years due to its potential therapeutic effects in treating multiple ailments while exhibiting minimal to no psychoactive properties. CBD has been reported to exhibit various effects on the human body. Studies suggest that it possesses anti-inflammatory, analgesic (pain-relieving), anxiolytic (anti-anxiety), and neuroprotective properties [60]. CBD has also shown potential in the treatment of epilepsy, schizophrenia, and other psychiatric disorders [61]. Additionally, it may have antioxidant and anticancer properties, through studies hypothesizing the mechanisms that CBD might enact on [62].

The mechanisms through which CBD exerts its effects are complex and multifaceted. CBD interacts with several molecular targets in the body, including cannabinoid receptors (CB1 and CB2), serotonin receptors (5-HT1A), and transient receptor potential (TRP) channels [63]. However, CBD does not bind strongly to these receptors, and its effects are believed to be largely mediated through the indirect modulation of endogenous neurotransmitter systems. CBD’s interaction with the endocannabinoid system (ECS) is of particular importance. Although CBD has low affinity for cannabinoid receptors, it can influence the ECS by inhibiting the enzyme fatty acid amide hydrolase (FAAH), which is responsible for the breakdown of anandamide, an endogenous cannabinoid. By inhibiting FAAH, CBD increases anandamide levels, leading to potential therapeutic effects [64]. Furthermore, CBD has been found to modulate various signaling pathways and molecular targets involved in inflammation, oxidative stress, and neurotransmission. It affects the release and uptake of neurotransmitters such as serotonin, dopamine, and glutamate, contributing to its anxiolytic and antipsychotic properties [65].

Considering the therapeutic applications of CBD and its low toxicity, a marked interest has emerged in the design of new analogs of hydrogenated bicyclic CBD and quinones [66] to study its pharmacological and clinical effects.

### 3.1. Hydrogenation of CBD and Its Derivatives

**Scheme 16:**
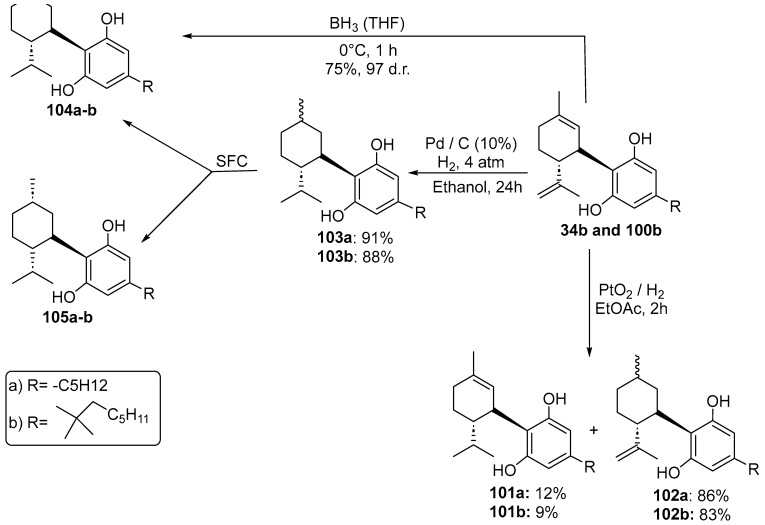
Partial and full hydrogenation of CBD (**34b**) and CBD-DMH (**100b**).

Ben-Shabat [9] reported the partial hydrogenation of CBD (**34b**) and dimethyl-cannabidiol (CBD-DMH, **100**) using Adam’s catalyst (PtO_2_) to afford a mixture of (1′*R*,2′*S*)-2′-isopropyl-5′-methyl-4-pentyl-1′,2′,3′,4′-tetrahydro-[1,1′-biphenyl]-2,6-diol (**101a**) from CBD or (1′R,2′S)-2′-isopropyl-5′-methyl-4-(2-methyloctan-2-yl)-1′,2′,3′,4′-tetrahydro-[1,1′-biphenyl]-2,6-diol (**101b**) from CBD-DMH (propen hydrogenated position), and 2-((1*S*,2*R*)-5-methyl-2-(prop-1-en-2-yl)cyclohexyl)-5-pentylbenzene-1,3-diol (**102a**) from CBD and 2-((1*S*,2*R*)-5-methyl-2-(prop-1-en-2-yl)cyclohexyl)-5-(2-methyloctan-2-yl)benzene-1,3-diol (**102b**) from CBD-DMH (C-5′ hydrogenated position), being products **102a** and **102b** of the predominant epimers (86% and 83%, respectively) (Scheme 16).

Also, Cruces et al. [27,28] described the full hydrogenation of CBD using Pd/C (10%) and hydrogen under 4 atm of pressure to obtain the racemic mixture of dihydro-CBD (**103a**) (Scheme 16).

Hydrogenated CBD analogs are relatively obscure compounds. Limited data and experiments have been conducted on the compound. Up until 2023, dihydro-CBD enantiomers (**104a** and **105a**) were not characterized, until earlier this year when Cruces et al. [10] successfully separated the pure enantiomers of dihydro-CBD (2-((1′*S*,2′*S*,5′*R*)-2-isopropyl-5-methylcyclohexyl)-5-alkylbenzene-1,3-diol, **104a** and 2-((1′*S*,2′*S*,5′*S*)-2-isopropyl-5-methylcyclohexyl)-5-alkylbenzene-1,3-diol, **105a**) with supercritical fluid chromatography (SFC) using a chiral column. The stereochemistry of the isomers was characterized using a combination of 1D and 2D NMR techniques and the purity was obtained using HPLC. The (*R*) and (*S*) isomers look similar, and there is a remarkable difference in their three-dimensional structures due to the change in stereochemistry of the cyclohexane/terpene ring. This difference in 3D shapes strongly suggests that one of the isomers could be far more active, interacting with the binding targets with increased affinity and specificity.

Marson [49] described the enantioselective catalytic hydrogenation of CBD using borane in THF to obtain the *R* enantiomer of dihydro-CBD (2-((1*S*,2*S*,5*R*)-2-isopropyl-5-methylcyclohexyl)-5-pentylbenzene-1,3-diol, **104a**) with 97% dr (Scheme 16).

### 3.2. Machaeridiols and Their Synthetic Analogs

Natural machaeridiol compounds have the skeleton configuration at 1*R* and 2*R* positions opposite to those at 1*S* and 2*S* positions of dihydro-CBD and the same as the enantiomer 5*S* position. Also, the machaeridiol chemotype is like dihydro-CBD, with the n-alkyl moiety replaced by steryl and benzofuranyl forms. HHDBP-type phytocannabinoids displayed potent activity against *Staphylococcus aureus* (vancomycin-resistant), *Enterococcus faecium*, and *E. faecalis* such as machaeridiols A [67,68]. The biological activities and interesting structural design of this class of natural compounds have inspired synthetic efforts directed toward their total syntheses.

**Scheme 17:**
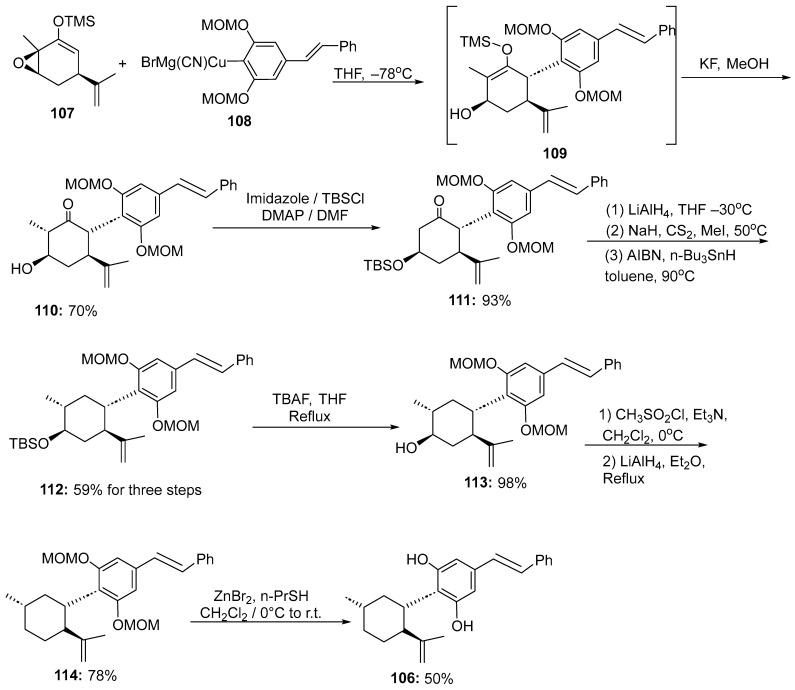
Ten-step synthetic pathway using various reagents to obtain (+) macheridiol A (**106**) developed by Huang [67].

Huang [67] introduced the first ten-step effective route for the synthesis of (+) machaeridiol A (**106**) using the regio- and stereoselective SN2′-reaction between trimethyl(((1*R*,4*S*,6*R*)-1-methyl-4-(prop-1-en-2-yl)-7-oxabicyclo[4.1.0]hept-2-en-2-yl)oxy)silane (**107**) and arylcyanocuprates (**108**) to obtain an adduct (**109**), which was hydrolyzed in situ to yield (2*R*,3*R*,5*R*,6*S*)-2-(2,6-bis(methoxymethoxy)-4-((*E*)-styryl)phenyl)-5-hydroxy-6-methyl-3-(prop-1-en-2-yl)cyclohexanone (**110**) with the four stereocenters that appear in the machaeridiol core. The second step was the protection of the hydroxyl group with tert-butyldimethylsilyl (TBS) to generate compound **111,** which underwent the reduction reaction with the use of lithium aluminum hydride (LiAlH_4_) followed by the xanthation process and reduction via Barton radical deoxygenation to afford compound **112**. For removing the hydroxyl group from the hexyl ring, first, it was deprotected and then treated with methanesulfonyl chloride to convert **113** into the corresponding methyl sulfonate derivative and reduce it with LiAlH_4_ to furnish 1,3-bis(methoxymethoxy)-2-((1*R*,2*R*,5*S*)-5-methyl-2-(prop-1-en-2-yl)cyclohexyl)-5-((*E*)-styryl)benzene (**114**). Finally, the deprotection of methoxymethyl (MOM) ethers using zinc bromide and propanethiol was carried out to obtain (+) machaeridiol A (**106**) with a 20% overall yield (Scheme 17).

**Scheme 18:**
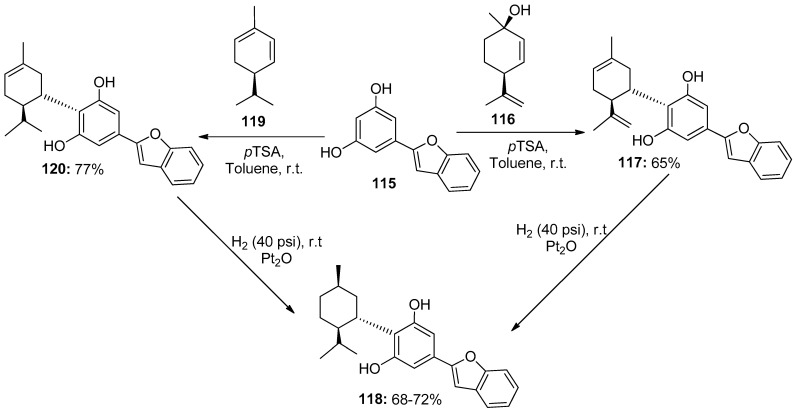
Synthesis of 5-(benzofuran-2-yl)-2-((1*S*,2*R*,5*R*)-2-isopropyl-5 methylcyclohexyl) benzene-1,3-diol (**118**).

Based on the 5-methyl-2-(prop-1-en-2-yl)cyclohexyl)benzene-1,3-diol (Figure 3, **46**) scaffold, Muhammad [68] obtained the machaeridiol analog (5-(benzofuran-2-yl)-2-((1*S*,2*R*,5*R*)-2-isopropyl-5-methylcyclohexyl)benzene-1,3-diol (**118**) via the coupling reaction between monoterpene units, (1*R*,4*S*)-1-methyl-4-(prop-1-en-2-yl)cyclohex-2-enol (**116**) or (*R*)-5-isopropyl-2-methylcyclohexa-1,3-diene (**119**) with 5-(benzofuran-2-yl)benzene-1,3-diol (**115**), followed by stereoselective reduction using Adam’s catalyst (Scheme 18).

## 4. Non-Classic Hydrated Phytocannabinoids and Their Synthetic Analogs

### 4.1. Cannabielsoin: A Metabolite of Cannabidiol

Research into non-classic saturated phytocannabinoids is growing rapidly. For example, (5a*S*,6*S*,9*R*,9a*R*)-6-methyl-3-pentyl-9-(prop-1-en-2-yl)-5a,6,7,8,9,9a-hexahydro dibenzo[*b*,*d*]furan-1,6-diol (CBE, **121**) has been reported as a CBD metabolite from plants and mammals and classified as non-classical cannabinoids for the modification of ring B (five-ring instead of six-ring) and in the northern aliphatic group in the CBD core. Furthermore, 1-((1*R*,3*S*,3a*S*,8b*R*)-8-hydroxy-6-pentyl-1-(prop-1-en-2-yl)-2,3,3a,8b-tetrahydro-1*H*-cyclopenta [b]benzofuran-3-yl)ethanone (anhydrocannabimovone: ACBM, **122**) and 1-((1*R*,2*R*,3*R*,4*R*)-3-(2,6-dihydroxy-4-pentylphenyl)-2-hydroxy-4-(prop-1-en-2-yl)cyclopentyl)ethanone (cannabimovone: BM, **123**) have been isolated from a strain of *Cannabis sativa*, but with very low percentages due to limited abundance in the plant and unmanageable purification and isolation processes [69]. However, no pharmacological studies of these non-classic hydrogenated cannabinoids have been carried out.

**Scheme 19:**
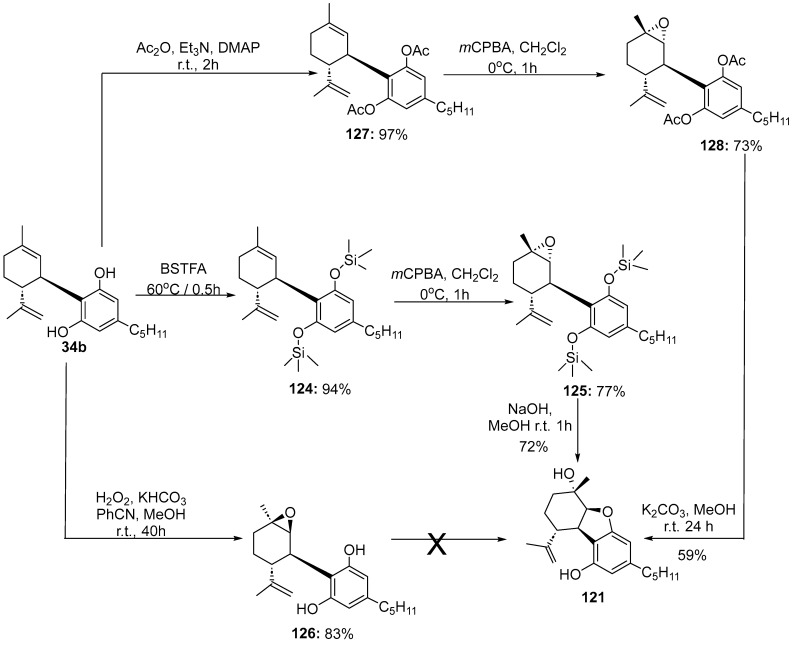
Synthesis of (5a*S*,6*S*,9*R*,9a*R*)-6-methyl-3-pentyl-9-(prop-1-en-2-yl)-5a,6,7,8,9,9a-hexahydro dibenzo[b,d]furan-1,6-diol (CBE, **121**) via epoxidation.

Williamson [70] and later Sarlah [71] developed different ways to synthesize CBE, **121** starting with CBD (**34b**). On the first route, CBD was completely silylated using *N*,*O* bis(trimethylsilyl)trifluoroacetamide (BSTFA) followed by chemoselective epoxidation to obtain 1*R*,2*R*,3*R*,6S-silyl epoxide (**125**), which was deprotected in the presence of sodium hydroxide in methanol to achieve CBE (**121**) in a 52% yield (Scheme 19). In the second way, the CBD underwent full acetylation and then chemoselective oxidation to give 1*R*,2*R*,3*R*,6S-acetyl epoxide (**128**). Epoxide **128** was exposed to an excess of potassium carbonate in methanol to deliver CBE (**121**) with a 42% overall yield. Williamson [70] carried out the epoxidation without protecting the CBD, which reversed the major facial selectivity of the epoxidation to obtain 1*S*,2*R*,3*R*,6*R*-epoxide (**126**) in an 83% yield. However, the cyclization of epoxide **126** to generate CBE failed (Scheme 19). It is due to a higher energy barrier for the equatorial attack of bases on cyclohexane-derived epoxides [72].

### 4.2. Cannabimovone, Anhydrocannabimovone, and Their Non-Natural Analogs

**Scheme 20:**
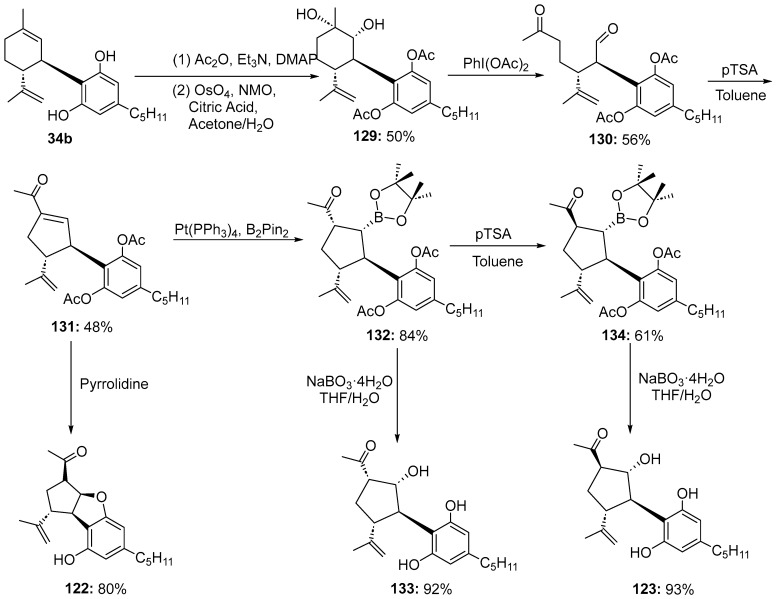
Synthetic pathway proposed by Sarlah [71] and Echavarren [73] to obtain the natural products ACBM (**122**) and *R*-CBM (**123**) and the synthetic diastereomer *S*-CBM (**133**).

ACBM (**122**) was found to be active against metabotropic and ionotropic cannabinoid receptors, displaying a similar biological outline to THC; however, *S*-CBM (**123**) has affinity just for ionotropic receptors [73]. Thinking of this pharmacological activity, Sarlah [71] and Echavarren [73] described a method to obtain *R*-CBM (**123**), its unnatural epimer, *S*-CBM (**133**), and ACBM (**122**) commencing from the full acetylation of CBD as it shows in Scheme 20. Osmium tetroxide (OsO_4_) was used as an oxidant in the dihydroxylation of the cyclohexyl ring on the AcO-CBD to produce syn-diol **129**, which was subjected to 1,2 diol cleavage using Phenyliodine(III)diacetate (PhI(OAc)_2_) to afford 2-((2*R*,3*R*)-1,6-dioxo-3-(prop-1-en-2-yl)heptan-2-yl)-5-pentyl-1,3-phenylene diacetate (**130**). After that, aldol condensation in the presence of *p*-toluene sulfonic acid allowed for obtaining 2-((1*R*,5*R*)-3-acetyl-5-(prop-1-en-2-yl)cyclopent-2-en-1-yl)-5-pentyl-1,3-phenylene diacetate (**131**). Finally, upon the acetyl group removal and intramolecular oxa-Michael reaction, ACBM (**122**) was generated with 2:1 dr and a 22% overall yield (Scheme 20).

*R*- and *S*-CBM (**123** and **133**) epimers were synthesized beginning with **131** via [Pt(PPh_3_)_4_]-catalyzed diboration using bis(pinacolato)diboron (B_2_Pin_2_) to introduce the boryl moiety, enantioselectively, in its structure and then to have boronate ester **132** undergo an oxidation with sodium perborate to furnish 1-((1*S*,2*R*,3*R*,4*R*)-3-(2,6-dihydroxy-4-pentylphenyl)-2-hydroxy-4-(prop-1-en-2-yl)cyclopentyl)ethanone (**133**) with a 19% overall yield. Boronate ester **132** was epimerized using *p*-toluenesulfonic acid to generate the mixture of diastereoisomers (5:1, dr), which was separated to give a 61% yield of 2-((1*R*,2*R*,3*R*,5*R*)-3-acetyl-5-(prop-1-en-2-yl)-2-(4,4,5,5-tetramethyl-1,3,2-dioxaborolan-2-yl)cyclopentyl)-5-pentyl-1,3-phenylenediacetate (**134**). After boronic ester **134** oxidation, CBM (**123**) was delivered with 11% overall yields on the seven-step synthetic route from commercially available CBD (**34b**) (Scheme 20).

## 5. Saturated Quinonoid Cannabinoid

There are various other saturated cannabinoids that have been explored and studied. Some of which include a variety of quinol compounds. Quinones have various biological activities and several natural and synthetic quinone compounds are currently used as therapeutic drugs. One particular cannabinoid quinone (HU-331: (1′*S*,6′*R*)-6-hydroxy-3′-methyl-4-pentyl-6′-(prop-1-en-2-yl)-[1,1′-bi(cyclohexane)]-2′,3,6-triene-2,5-dione) was synthesized in 1968 to address the question of cannabinoids giving a purple color extracted with 5% aqueous KOH in methanol (Beam Test) [74,75]. Much later in the 1990s, HU-331 was studied once again due to the dual potential of its anticancer quinone moiety and non-toxic cannabinoid function. Cannabinoids have distinct pharmacokinetic properties when compared to the known quinoid anticancer drugs. HU-331 was shown to have very high efficacy against human cancer cell lines in vitro and against in vivo grafts of human tumors in nude mice [66,75,76,77]. Although HU-331 is not saturated, there are several hydrogenated derivates such as 1′*R*,6′*S*)-6-hydroxy-6′-isopropyl-3′-methyl-4-pentyl-[1,1′-bi(cyclohexane)]-2′,3,6-triene-2,5-dione (**135**), (1′*S*,2′*R*)-6-hydroxy-5′-methyl-4-pentyl-2′-(prop-1-en-2-yl)-[1,1′-bi(cyclohexane)]-3,6-diene-2,5-dione (**136**), (6a*R*,10a*S*)-6,6,9-trimethyl-3-pentyl-6a,7,8,9,10,10a-hexahydro-1*H*-benzo[c]chromene-1,4(6*H*)-dione (**137**), (6a*R*,10a*S*)-6,6,9-trimethyl-3-pentyl-6a,7,8,9,10,10a-hexahydro-1*H*-benzo[c]chromene-1,2(6*H*)-dione (**138**), (6a*R*,10a*S*)-8-hydroperoxy-6,6-dimethyl-9-methylene-3-pentyl-6a,7,8,9,10,10a-hexahydro-1*H*-benzo[c]chromene-1,4(6*H*)-dione (**139**), and (6a*R*,8*R*,10a*S*)-8-hydroxy-6,6-dimethyl-9-methylene-3-pentyl-6a,7,8,9,10,10a-hexahydro-1*H*-benzo[c]chromene-1,4(6*H*)-dione (**140**).

### 5.1. Different Oxidation Pathways of Hydrogenated Cannabidiol and Tetrahydrocannabinol Derivatives

**Scheme 21:**
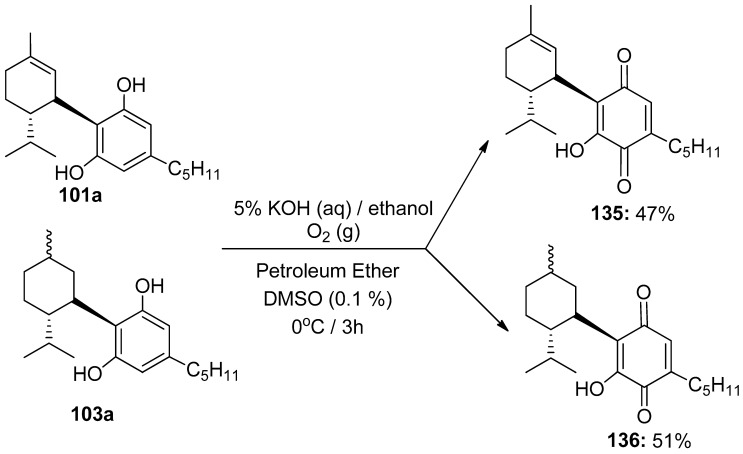
Oxidation of H_2_-CBD (**101a**) and H_4_-CBD (**103a**) to obtain their corresponding quinone derivatives in the presence of oxygen.

Kogan [61] synthesized (1′*R*,6′*S*)-6-hydroxy-6′-isopropyl-3′-methyl-4-pentyl-[1,1′-bi(cyclohexane)]-2′,3,6-triene-2,5-dione (**135**) and (1′*S*,2′*R*)-6-hydroxy-5′-methyl-4-pentyl-2′-(prop-1-en-2-yl)-[1,1′-bi(cyclohexane)]-3,6-diene-2,5-dione (**136**) with around a 50% yield from H_2_CBD and H_4_CBD, respectively, using an aqueous potassium hydroxide (5%) solution in ethanol and bubbling the O_2_ into the reaction mixture (Scheme 21).

**Scheme 22:**
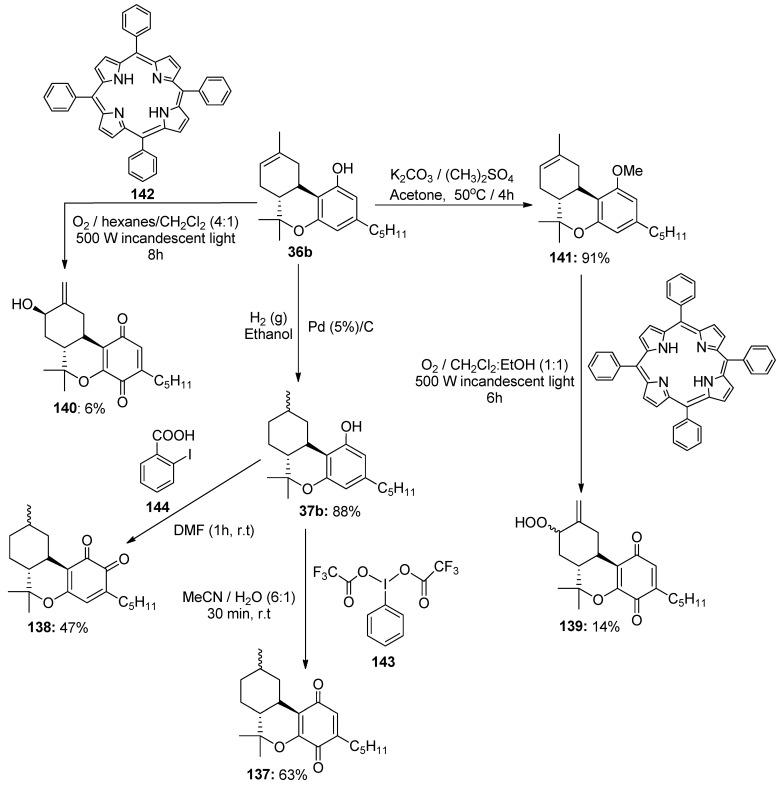
Different oxidation pathways of HHC derivatives to obtain HHC-quinones.

In 2018, El Sohly’s team [78] reported the synthesis of cannabinoid–quinones (**139** and **140**) based on tricyclic HHC. The introduction of the *p*-quinone core was carried out by the irradiating with 500 W incandescent light of THC analogs (**141** and **36b**) in the presence of 5,10,15,20-tetraphenyl-21*H*,23*H*-porphyrin and O_2_. (6a*R*,10a*S*)-8-hydroperoxy-6,6-dimethyl-9-methylene-3-pentyl-6a,7,8,9,10,10a-hexahydro-1*H*-benzo[c]chromene-1,4(6*H*)-dione (**139**) and (6a*R*,8*R*,10a*S*)-8-hydroxy-6,6-dimethyl-9-methylene-3-pentyl-6a,7,8,9,10,10a-hexahydro-1*H*-benzo[c]chromene-1,4(6*H*)-dione (**140**) were afforded with very low yields, 14% and 6%, respectively (Scheme 22).

On the other hand, Deng [79] and Morales [80] carried out the oxidation of the HHC racemic mixture (**37b**) in the presence of two different oxidizing agents. When (bis(trifluoroacetoxy)iodo)benzene was used, in an air open container, *para* (6a*R*,10a*S*)-6,6,9-trimethyl-3-pentyl-6a,7,8,9,10,10a-hexahydro-1*H*-benzo[c]chromene-1,4(6*H*)-dione (**137**) was accomplished. However, when using 2-iodobenzoic acid, (6a*R*,10a*S*)-6,6,9-trimethyl-3-pentyl-6a,7,8,9,10,10a-hexahydro-1*H*-benzo[c]chromene-1,2(6*H*)-dione (**138**) was afforded.

### 5.2. Applying the Domino Knoevenagel Intramolecular Hetero Diels–Alder Reaction to Obtain Benzoquinone Derivatives

Aside from cannabinoid-specific quinones, there are countless other quinone scaffolds that could also be applied to the cannabinoid core. Estévez-Braun [81] discusses a series of chromene–benzoquinone derivatives that were synthesized through the *one-pot* domino Knoevenagel intramolecular hetero Diels–Alder reaction starting with 2,5-dihydroxy-3-undecylcyclohexa-2,5-diene-1,4-dione (**145**) and unsaturated aldehydes (**9a**, **151**, and **154**). 2,5-dihydroxy-3-undecylcyclohexa-2,5-diene-1,4-dione is a natural product isolated as an active ingredient from the *Embelia ribes* plant [82]. It is an interesting scaffold because it has exhibited anti-inflammatory [83,84,85], antibacterial [86,87], antitumor [88,89], and anticonvulsant [90] effects.

**Scheme 23:**
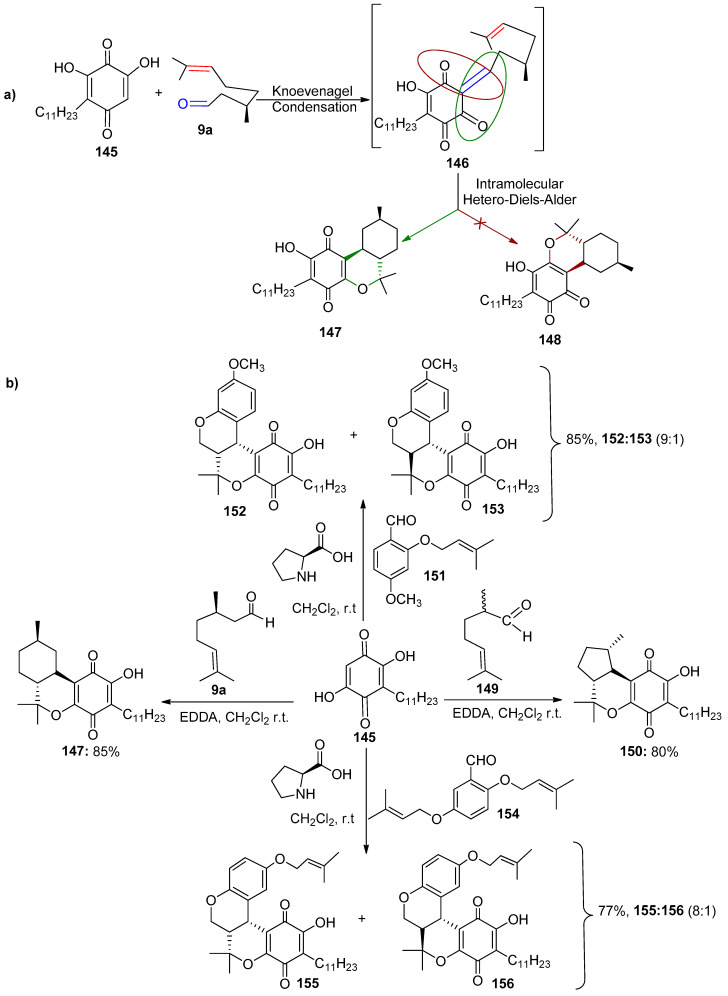
Reaction between 2,5-dihydroxy-3-undecylcyclohexa-2,5-diene-1,4-dione (**145**) and unsaturated aldehydes to obtain cannabinoid–quinone analogs. (**a**) depicts the reaction mechanism, Condensation reaction depicts in blue the bond created, with the green circle depicting the cyclization proceeding, while the brown circle depicts the inability for the cyclization to occur. (**b**) depicts the transformation of CBD-quinone to various analogs.

The coupling reaction between 2,5-dihydroxy-3-undecylcyclohexa-2,5-diene-1,4-dione (**145**) and (*R*)-3,7-dimethyloct-6-enal (**9a**), where the keto group is close to a double bond, drives to the formation of adduct **146,** which suffers, in situ, an intramolecular hetero Diels–Alder reaction with the dienophile moiety, affording the corresponding chromene–benzoquinone derivatives (**147** and **148**). Polyfunctional adduct **146** has two possible dienes to combine with the dienophile that could lead to *ortho*- or *para*-benzoquinonic derivatives (**147** and **148**); however, only the **147** diastereomer was obtained. The high diastereoselectivity through the intramolecular hetero Diels–Alder reaction can be interpreted because the exo-*E*-anti transition state is the only one that can be formed since the endo-*Z*-anti transition state has a geometric impediment to be reached (Scheme 23a).

Some cannabinoid–quinone analogs were accomplished using this approach to study the biological and selective activity against Gram-positive bacteria, including resistant *Staphylococcus aureus* isolated from a hospital. Knoevenagel condensation was carried in the presence of different organic catalysts such as 1,2-ethanediamine acetate (EDDA) or (*S*)-pyrrolidine-2-carboxylic acid. For the synthesis of (6a*R*,9*R*,10a*S*)-2-hydroxy-6,6,9-trimethyl-3-undecyl-6a,7,8,9,10,10a-hexahydro-1*H*-benzo[c]chromene-1,4(6*H*)-dione (**147**) and (1*S*,3a*R*,9b*S*)-8-hydroxy-1,4,4-trimethyl-7-undecyl-1,2,3,3a,4,9b-hexahydrocyclopenta[*c*]chromene-6,9-dione (**150**), the best results were obtained using EDDA in dichloromethane. However, when unsaturated aromatic aldehydes (**151** and **154**) were employed to form tetracyclic chromene–benzoquinone derivatives, EDDA gave poor diastereoselectivity, obtaining a racemic mixture of *cis* and *trans* compounds (**152**/**153** and **155**/**156**). With the objective of improving the diastereomeric rate, they implemented the condensation in the presence of (*S*)-pyrrolidine-2-carboxylic acid, a chiral amino acid, and under these conditions, the *cis* diastereomer was obtained in a higher ratio (**152:153**, 9:1 and **155:156**, 8:1).

## 6. Bi-, Tri-, and Tetra-Cyclic Hydrogenated Natural Cannabinoid Scaffolds

**Figure 4:**
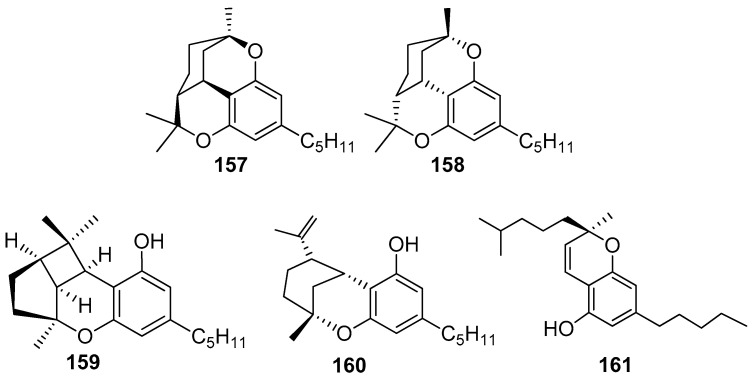
Structures of (−) cannabicitran (**157**), (+)-cannabicitran (**158**), cannabicyclol (**159**), Δ8-*iso*-*cis*-THC (**160**), and tetrahydrocannabichromene (**161**).

Cannabichromene, (*R*)-2-methyl-2-(4-methylpent-3-en-1-yl)-7-pentyl-2*H*-chromen-5-ol (CBC, **163**), is a minor, chiral, non-psychoactive cannabinoid found in *Cannabis Sativa*. Since first being isolated and identified in the 1960s from hashish oil, studies have shown CBC to be a powerful and potent selective CB2 and TRPA1 agonist, leading to its anti-inflammatory activity [91]. CBC is the starting point for obtaining various bi-, tri-, and tetra-cyclic hydrogenated natural cannabinoid scaffolds such as (6a*R*,9*S*,10a*S*)-6,6,9-trimethyl-3-pentyl-6a,7,8,9,10,10a-hexahydro-6*H*-1,9-epoxybenzo[c]chromene (**158**), (1a*R*,1a1*S*,3a*S*,8b*S*)-1,1,3a-trimethyl-6-pentyl-1a,1a1,2,3,3a,8b-hexahydro-1*H*-4-oxabenzo[*f*]cyclobuta[*cd*]inden-8-ol (**159**), (2*R*,5*S*,6*R*)-2-methyl-9-pentyl-5-(prop-1-en-2-yl)-3,4,5,6-tetrahydro-2*H*-2,6-methanobenzo[*b*]oxocin-7-ol (**160**), and (*R*)-2-methyl-2-(4-methylpentyl)-7-pentyl-2*H*-chromen-5-ol (**161**) (Figure 4).

### 6.1. Cannabicitran

**Scheme 24:**
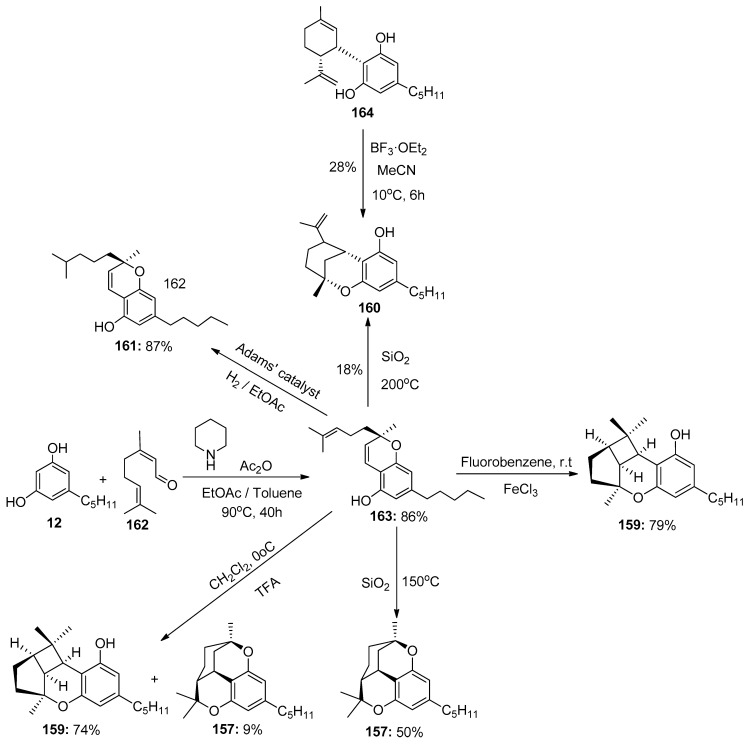
Synthetic procedure to obtain (−) cannabicitran (**157**), cannabicyclol (**159**), Δ8-*iso*-*cis*-THC (**160**), and tetrahydrocannabichromene (**161**).

Cannabicitran (CBT, **157**/**158**) is another naturally found hydrogenated cannabinoid that is saturated and epoxide-containing. Recently, Williamson [92] demonstrated that CBT appears as a racemic mixture in a *Cannabis sativa* plant after separating both enantiomers: (6a*S*,9*R*,10a*R*)-6,6,9-trimethyl-3-pentyl-6a,7,8,9,10,10a-hexahydro-6*H*-1,9-epoxybenzo[c]chromene (**157**) and (6a*R*,9*S*,10a*S*)-6,6,9-trimethyl-3-pentyl-6a,7,8,9,10,10a-hexahydro-6*H*-1,9-epoxybenzo[c]chromene (**158**) via preparative HPLC chromatography using a chiral column (Figure 4).

(−)-CBT was synthesized via the [3 + 3] Knoevenagel annulation between olivetol (**12**) and (*Z*)-3,7-dimethylocta-2,6-dienal (**161**) affording (*R*)-7-butyl-2-methyl-2-(4-methylpent-3-en-1-yl)-2*H*-chromen-5-ol (**162**), which suffered an acid–catalyst intramolecular [2 + 2] cyclization in the presence of silica gel [93] or trifluoroacetic acid [94,95] to yield (6a*S*,9*R*,10a*R*)-6,6,9-trimethyl-3-pentyl-6a,7,8,9,10,10a-hexahydro-6*H*-1,9-epoxybenzo[c]chromene (**157**) with 50% or 9%, respectively (Scheme 24).

### 6.2. Cannabicyclol

Another saturated natural cannabinoid is cannabicyclol (CBL, **159**). CBL’s structure had several revisions until finally Marlowe [96] established, with an X-ray analysis, (1a*R*,1a^1^*S*,3a*S*,8b*S*)-1,1,3a-trimethyl-6-pentyl-1a,1a^1^,2,3,3a,8b-hexahydro-1*H*-4-oxabenzo[*f*]cyclobuta[cd]inden-8-ol (**159**) as the absolute configuration of CBL after treating it with (*S*)-(+)-ibuprofen (Figure 4).

CBL (159) was synthesized by Hsung [97] with 74% of yield from (*R*)-7-butyl-2-methyl-2-(4-methylpent-3-en-1-yl)-2*H*-chromen-5-ol (162) via cationic [2π + 2π] cyclization in the presence of trifluoracetic acid in dichloromethane at 0 °C. CBT (157) was formed as a byproduct with only 9%. Later, Li [98] developed a pathway to obtain CBL from compound 162 using FeCl_3_ in fluorobenzene with a 79% yield and 0% of CBT (157) (Scheme 24).

### 6.3. Δ8-Iso-Cis-THC

Δ8-*iso*-*cis*-THC (**160**) is obtained with 18% from the protonation of the ald.rhatic double bond in (*R*)-7-butyl-2-methyl-2-(4-methylpent-3-en-1-yl)-2*H*-chromen-5-ol (**162**) followed by the formation of a benzylic cation, and finally enclosed by the terminal 2-methylbut-2-ene double bond and the loss of a proton [93] (Scheme 20). Also, it can be accomplished starting with (1′*S*,2′*R*)-5′-methyl-4-pentyl-2′-(prop-1-en-2-yl)-1′,2′,3′,4′-tetrahydro-[1,1′-biphenyl]-2,6-diol (**163**) using boron trifluoride etherate as an acid–catalyst and acetonitrile as a solvent at −10 °C via cyclization from the Δ5′ double bond (Scheme 24).

### 6.4. Tetrahydrocannabichromene

Gaoni [94] reported the synthesis of tetrahydrocannabichromene ((*R*)-2-methyl-2-(4-methylpentyl)-7-pentyl-2*H*-chromen-5-ol: **161**) via the catalytic hydrogenation of CBC (**163**) using Adam’s catalyst (PtO_2_·H_2_O) at the atmospheric pressure of hydrogen. Compound **161** was afforded with 87% of yield.

## 7. Biological Studies of Saturated Cannabinoids

Although saturated cannabinoids have been known for about 100 years, no absorption, distribution, metabolism, and excretion (ADME) studies have been published in peer-review journals. It is important to consider that HHC has invaded the recreational market in the last 2 years and its consumption by inhaling, ingesting in the form of edibles, or taking it sublingually with oils could trigger psychotropic effects by not knowing the proper dosages and side effects of this product and its analogs.

For this reason, research on the mechanism of action, the interaction in the human organism, and the new biological applications of HHCs and their analogs should be a priority in research projects.

**Figure 5:**
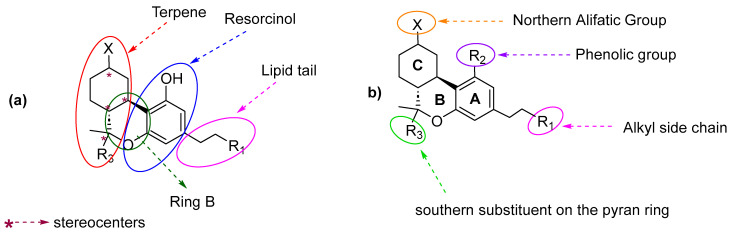
(**a**) HHC scaffold broken down into five distinct regions. (**b**) Four major pharmacophores present in the HHC core.

In this section of the review, we compiled all the data on the affinities of saturated cannabinoids for CB1 and CB2 receptors and their relationship with the different functionalities in the HHC scaffold, considering the five distinct regions (terpene moiety, ring B, resorcinol core, lipid tail, and stereocenters) or the four main pharmacophores (alkyl side chain, phenolic hydroxyl group, northern aliphatic group, and southern substituent in the pyran ring) in the HHC structure, which are important for cannabimimetic receptor affinities (Figure 5).

The modification in the terpene moiety determines the role of the ring rigidity and whether the introduction of hydrogen bond donors and acceptors could influence the affinity and selectivity for both CB1 and CB2 receptors. The alteration of the resorcinol ring allows for examining the effect of the free hydroxyl group, protecting forming ethers, oxidizing forming quinones, or removed on the biological activity of hydrogenated cannabinoids. The alkyl chain and stereocenters permit to an evaluation of how geometric constraints and lipophilicity influence binding pockets. Finally, it is important to determine the difference between bicyclic cannabinoids (CBD analogs, ring B opened) and tricyclic cannabinoids (THC analogs, ring B closed) in receptor affinity.

The search to comprehend the molecular basis of the pharmacological effects of cannabinoids led to the identification and characterization of CB receptors. The cannabinoid receptors are membrane-bound receptors that belong to a superfamily of G-protein coupled receptors (GPCRs). To date, two CB receptors, CB1 and CB2, have been isolated, cloned, and expressed. The first cannabinoid receptor (CB1) was discovered when Matsuda cloned and expressed this GPCR from rat brains in 1990 [99] followed by the expression of human CB1 in 1991 by Gerard [100]. In 1993, Munro found, cloned, and expressed a second cannabinoid receptor (CB2) within the preparation of a human promyelocytic leukemia cell line (HL60) [101].

### 7.1. In Vitro Studies to Determine Affinities of Hydrogenated Cannabinoids for CB1 and CB2 Receptors

In contrast to CBD (**34b**), 2-((1*S*,2*S*)-2-isopropyl-5-methylcyclohexyl)-5-pentylbenzene-1,3-diol (**103a**, Table 2) and 2-((1*S*,2*S*)-2-isopropyl-5-methylcyclohexyl)-5-(2-methyloctan-2-yl)benzene-1,3-diol (**103b**, Table 2) have affinity for the cannabinoid CB1 receptor. It means that by removing the double bond from ring C and from the southern aliphatic chain, the ability to bind to the CB1 receptor increases. Also, by branching the lipophilic chain incorporating two methyl groups, the affinity for the CB1 receptor (comparing compounds **103a** and **103b**) was improved. Ben-Shabat [9] demonstrated that the anti-inflammatory capacity of these compounds owes its origin to the effect on the production of reactive oxygen intermediates (ROIs), nitric oxide (NO), and tumor necrosis factor (TNF). Moreover, Ben-Shabat [9] concluded that the activation of such mediators is not directly through central cannabinoid receptor CB1 because compound **103b** showed decreased suppressive effects on ROI, NO, and TNF-R production compared to compound **103a** (Table 2).

Macheriols and machaeridiols are important types of hexahydrodibenzopyran-cannabinoids. Macheriols are characterized by having a chromane core and an ABC tricyclic system, structurally similar to HHC, and machaeridiols are defined by the open B pyran ring, which resembles H_4_CBD [102]. The main difference lies in the inversion of stereocenters on position 6a and 10a for machaeriol or 1 and 2 for machaeridiols. Also, these compounds showed an aralkyl group as a side chain instead of a lipophilic chain as HHC and H_4_CBD.

Thapa et al. [89] demonstrated that anticancer effects of novel machaeridiol and machaeriol analogs imply the inhibition of cell proliferation and tumor angiogenesis and recently, Muhammad et al. [102] examined the in vitro cytotoxicity of some natural macheriols and machaeridiols against human solid tumor cell lines such as SK-MEL, KB, BT-549, SK-OV-3, and HeLa. They confirmed that the combination (1:1) of compound **39** (macheriol B) and compound **167** (machaeridiol B) exhibited activity against the five human cancer cell lines with an IC_50_ between 26 and 33 μg/mL.

Table 3 reveals that machaeridiols A, B, and C (**106**, **167**, and **168**) show selective binding affinities for CB2 receptors; however, machaeriol C and D (**39** and **43**) exhibit affinities for both CB1 and CB2 receptors.

**Figure 6:**
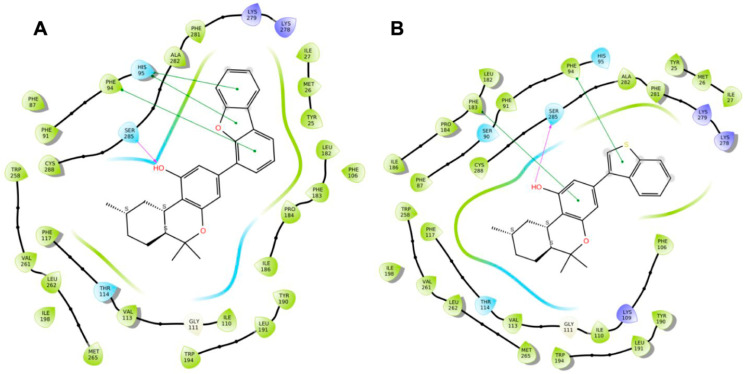
Two-dimensional interaction diagrams, with the green representing hydrophobic interactions, and the blue representing hydrophilic interactions. (**A**) Compound **166** against the CB1 receptor; (**B**) compound **166** against the CB2 receptor [103].

Chittiboyina et al. [103] designed a synthetic machaeriol (compound **166**, Table 3) that is a CB2-selective agonist, which is characterized by a benzothiophene moiety in the side chain. They performed in silico molecular docking experiments to explain the binding affinities of compound **166** into the active sites of CB1 and CB2 receptors’ protein crystal structures using Maestro, Schrödinger (Figure 6A). This compound showed π–π stacking interactions between hexahydrochromane and benzothiophene cores with the residues Phe170, Phe268, and Trp279 of the CB1 receptor. In addition, **166** generated hydrophobic interactions with a series of aquaphobic residues involving Phe108, Phe174, Phe177, Leu193, Val196, Phe200, Ile267, Trp279, Trp356, Leu359, Phe379, Ala380, and Cys386. In a similar fashion, compound **166** exhibited π–π stacking and hydrophobic interactions with CB2 residues. However, the major difference lay in the H-bonding shown between the hydroxyl group of the resorcinol ring and Ser285 (Figure 6B, marked with a purple circle), which is an essential residue for CB2 receptor activity.

We consider it essential to carry out a more in-depth study of SAR on machaeriol and machaeridiol derivatives to achieve novel analogs with better CB2 receptor selectivity, focusing on the side chain and the stereocenters of the HHDBP scaffold (**46**).

Table 4, Table 5 and Table 6 show how the four main pharmacophores influence the binding affinities of nonclassical and hybrid saturated tricyclic cannabinoids for CB1 and CB2 receptors in in vitro experiments and SAR studies.

### 7.2. Southern Aliphatic Hydroxyl Chain (SAH)

Modification of SAH generate a family of non-classical cannabinoids that have not been found in the cannabis plant [56,104,105]. First, we focus on the effect of the orientation of the SAH group. For this, Makriyannis [105] synthesized compounds **197** and **198**, demonstrating that the epimer (6*S*,6a*R*,9*R*,10a*S*)-6-(2-hydroxyethyl)-6-methyl-3-pentyl-6a,7,8,9,10,10a-hexahydro-6*H*-benzo[c]chromene-1,9-diol (**197**), with the hydroxyethyl group being in the equatorial position, has greater affinity for both receptors CB1 and CB2, resulting in more favorable ligand–receptor interaction (Table 4). Second, Makriyannis carried out SAR studies to examine the role of the hydroxyalkyl chain length and bulk in the activity of this scaffold. The binding affinities of compounds **188**, **189**, and **190** indicate little change in the CB1 and CB2 receptor affinity with increasing chain length. From the receptor binding data that display compounds **186**, **191**, and **190,** it can be concluded that the conformation of the side chain is not important for ligand–receptor interaction since the alkyne (**191**) and alkene (**186**) analogs exhibit similar receptor affinity to that of the hydroxyalkyl analog (**190**). When incorporating a halogen such as iodine (compound, **199**), the binding affinity for the CB1 and CB2 receptor decreases. From these results, it can be concluded that while the relative configuration 6-axial or 6-equatorial of the SAH appears to be critical, the length and the conformation of the southern hydroxyl chain are of lesser effect in determining the cannabinoid activity. Including a halogen atom is reflected in the loss of affinity for CB1 and CB2 receptors.

### 7.3. Northern Aliphatic Group (NAG)

Regarding NAG, we examined the role of the stereochemistry at C-10, the length of the C-10 substituent, and the functionality at C-10 in the cannabimimetic activity. The binding affinities’ data for CB1 and CB2 receptors appear in Table 4, Table 5 and Table 6. Table 6, which represents novel hydrogenated adamantyl cannabinoids, shows that all 10β-epimers (the equatorial orientation of the C-10-alkyl chain) improve CB1 and CB2 affinities compared to the 9*α*-epimers. The length of the C-10-alkyl chain does not affect the CB1 and CB2 affinities comparing compounds **219** and **224** in Table 6. The iodo-methyl derivative (**221**) sharply decreased CB1/CB2 affinities, revealing poor steroelectronic interactions at CB1 and CB2 residues. Judging by the data of binding affinities of pair compounds **217**/**224** (Table 6) and **93**/**89** (Table 4), the functionality on C-10 revealed a better CB1/CB2 affinity of CH_2_OH compared with OH. Judging by the data of binding affinities of pair compounds **217**/**224** (Table 6) and **93**/**89** (Table 4), C-10 functionality (CH_2_OH) revealed better CB1/CB2 than the OH group. In general, a hydroxyl group at the northern section of the tricyclic cannabinoids boosts the ligand’s affinity for both CB receptors. Contrasting the CB1/CB2 affinity value of compound **93**, Table 4 (3.0/2.1) and **179**, Table 4 (0.6/2.65), it proves that the introduction of the azido group (**179**) increases the affinity for the CB1 receptor and it remains the same (the affinity for the CB1 receptor).

### 7.4. Phenolic Group

Cannabinoid derivatives in which the hydroxyl group in the resorcinol core was removed or substituted by an alkyl chain to generate an ether group significantly decrease ligand binding to CB1, displaying better selectivity for the CB2 receptor (comparing compounds **200** and **201**, Table 5). Compound **201**, the corresponding methyl ether of **200**, exhibits more than 2000-fold CB2 selectivity. Interestingly, affinity to CB2 is only faintly altered by these changes.

### 7.5. Alkyl Side Chain

The manipulation of the electronics and conformational flexibility of the lipophilic side chain reveals the complexity and specificity of the cannabinoid-binding pocket as Table 4 and Table 5 show.

Ramification between C-1′ and C-2′ in the side chain specifically introducing a dimethyl or cyclopentyl group as shown in compounds **172**/**173**, **184**/**185**, and **181**/**185** leads to increased receptor affinity and selectivity, obtaining a CB1 receptor selective antagonist when it introduces a four-carbon cycle between C-1′ and C-2′ (compound **193**). Regarding unsaturation at the lipidic chain, no further increase in potency is noted when C-2′ and C-3′ are joined by a double bond, as illustrated in compound **206** (alkene) compared with **207** (unsaturated chain) or compound **182,** which has a double bond between C-1′ and C-2′ compared with **184** (alkane). However, in compound **181,** which presents a triple bond at C-1′ and C-2′, the CB2-biding affinity decreases relating to **182** (alkene) and **184** (alkane). The addition of a halogen group and the end of the side chain slightly affects the receptor affinity (compounds **193**, **195**, and **196**). Targeted covalent inhibitors (TCIs) represent an interesting development in cannabinoid ligands. Two major types of covalently activated lipidic chains have been employed as TCIs, those upholding electrophilic or photoactivatable functionality. For example, compounds **170**, **174**, and **177,** which have attached an azide (-N_3_, photoactivatable moiety), isothiocyanate (-NCS, electrophilic functional group), or cyano (-NC, electrophilic functional group) functionality, respectively, reduce the CB1 and CB2 receptor affinity (Table 4). Makriyannis [56] carried out molecular docking studies based on the hCB1 crystal structure (PDB: 5XR8). They explored the interactions of typical lipid-chain agonists with the CB1 receptor through molecular docking, revealing that all agonists adopt an L-shape configuration in the orthosteric-binding pocket. The interactions between the tricyclic HHC core system and CB1 are essentially hydrophobic and aromatic. For example, the π–π interactions with Phe268, Phe379, Phe189, and Phe177 residues and phenolic hydroxyl form a hydrogen bond with Ser383.

**Table 4:** Affinities (K*i*) of hybrid/non-classical cannabinoids for rCB1, hCB1, vv, and hCB2.

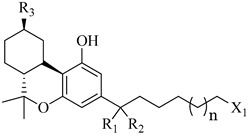							
**Compound**	**K*i* (nM)**					**Function**	**References**
	**rCB_1_**	**hCB_1_**	**rCB_2_**	**mCB_2_**	**hCB_2_**		
X_1_ = H, *n* = 2 R_1_ R_2_ 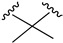 R_3_ = CH_2_OH **93**	3.0 ± 0.8	-	-	-	2.1 ± 0.6	Agonist	[56]
X_1_ = H, *n* = 2 R_1_ R_2_ 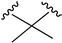 R_3_ = OH **89** (Canbisol)	19.0 ± 0.6				13.1 ± 0.2	-	[56]
X_1_ = N_3_, *n* = 2 R_1_ R_2_ 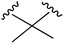 R_3_ = CH_2_OH **169**	0.41 ± 0.05	-	-	0.8 ± 0.1	1.4 ± 0.06	Agonist	[56]
X_1_ = N_3_, *n* = 2 R_1_ R_2_ 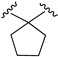 R_3_ = CH_2_OH **170**	0.40 ± 0.1	-	-	0.8 ± 0.1	0.8 ± 0.1	Agonist	[56]
X_1_ = N_3_, *n* = 3 R_1_ R_2_ 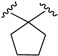 R_3_ = CH_2_OH **171**	0.5 ± 0.2	-	-	1.6 ± 0.1	1.5 ± 0.3	Agonist	[56]
X_1_ = NCS, *n* = 2 R_1_ R_2_ 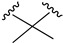 R_3_ = CH_2_OH **172**	0.39 ± 0.04		-	0.8 ± 0.1	3.15 ± 0.04	Agonist	[56]
X_1_ = NCS, *n* = 2 R_1_ = R_2_ = H R_3_ = CH_2_OH **173**	5.65 ± 0.1	9.0 ± 0.4	-		10.50 ± 0.02	Agonist	[56]
X_1_ = NCS, *n* = 2 R_1_ R_2_ 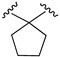 R_3_ = CH_2_OH **174**	1.1 ± 0.1	-	-	0.9 ± 0.2	1.3 ± 0.05	Agonist	[56]
X_1_ = NCS, *n* = 3 R_1_ R_2_ 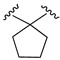 R_3_ = CH_2_OH **175**	0.4 ± 0.1	-	-	1.1 ± 0.1	1.0 ± 0.2	Agonist	[56]
X_1_ = CN, *n* = 2 R_1_ R_2_ 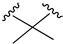 R_3_ = CH_2_OH **176**	0.4 ± 0.05	-	-	0.8 ± 0.1	0.4 ± 0.2	Agonist	[56]
X_1_ = CN, *n* = 2 R_1_ R_2_ 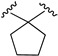 R_3_ = CH_2_OH **177**	0.8 ± 0.2	-	-	1.0 ± 0.1	1.4 ± 0.2	Agonist	[56]
X_1_ = CN, *n* = 3 R_1_ R_2_ 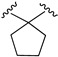 R_3_ = CH_2_OH **178**	0.5 ± 0.1	-	-	0.9 ± 0.1	0.4 ± 0.05	Agonist	[56]
X_1_ = N_3_, *n* = 2 R_1_ R_2_ 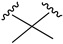 R_3_= N_3_ **179**	0.60 ±0.2	-	-	-	2.65 ±0.3	Agonist	[105]
X_1_ = I, *n* = 2 R_1_ R_2_ 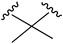 R_3_= N_3_ **180**	0.67 ± 0.1	-	-	-	0.72 ± 0.1	Agonist	[105]
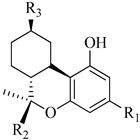							
**Compound**	**K*i* (nM)**					**Function**	**References**
	**rCB_1_**	**hCB_1_**	**rCB_2_**	**hCB_2_**	**mCB_2_**		
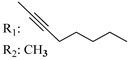 R_3_: CH_2_OH **181**	-	5.8	-	61.6	-	-	[105]
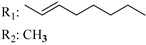 R_3_: CH_2_OH **182**	-	1.2	-	5.3	-	-	[105]
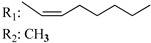 R_3_: CH_2_OH **183**	-	0.8	-	9.5	-	-	[105]
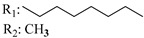 R_3_: CH_2_OH **184**	-	1.7	-	14.3	-	-	[105]
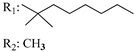 R_3_: CH_2_OH **185**		0.045		0.061			[105]
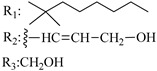 **186**		0.7		8.6			[105]
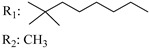 R_3_: OH **187**	-	2.3	-	2.3	-	-	[105]
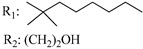 R_3_: CH_2_OH **188**	-	2.8	-	2.3	-	-	[105]
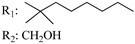 R_3_: CH_2_OH **189**	-	2.9	-	2.4	-	-	[105]
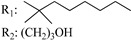 R_3_: CH_2_OH **190**	-	2.2	-	3.4	-	-	[105]
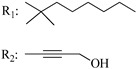 R_3_: CH_2_OH **191**	-	1.21	-	0.3	-	-	[105]
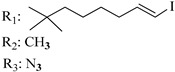 **192**	-	0.80	-	0.85	-	-	[105]
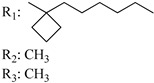 **193**	-	0.16	-	42.1	-	CB_1_ receptor selective antagonist	[106]
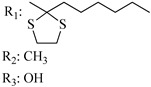 **194**	-	4.51 ± 0.7	-	13.9 ± 3.4	-	-	[56,57]
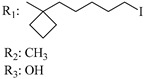 **195**	-	3.16 ± 0.05	-	4.21 ± 0.93	5.13 ± 1.27	-	[56,57]
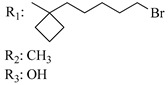 **196**	-	1.37 ± 0.35	-	2.76 ± 0.63	1.62 ± 0.45	-	[56,57]
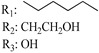 **197**	-	70.5	-	1.99	-	-	[105]
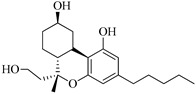 **198**	-	1353.9		2476.7	-	-	[105]
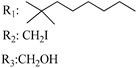 **199**		40.7		19.7			[56,57]

**Table 5:** Affinities (K*i*) of hybrid/non-classical cannabinoids for hCB1, mCB2, and hCB2.

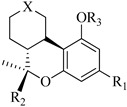							
**Compound**	**K*i* (nM)**					**Function**	**References**
	**rCB_1_**	**hCB_1_**	**rCB_2_**	**mCB_2_**	**hCB_2_**		
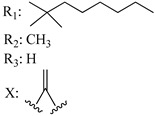 **200**	-	1.82	-	-	0.58	Agonist Mixed CB_1_/CB_2_	[105]
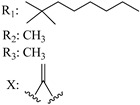 **201**	-	>20,000	-	-	1.94	CB_2_ Selective Agonist	[105]
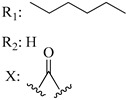 **202**	-	333.0	-	265	-	-	[56,57]
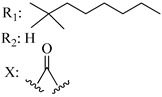 **88** (Nabilone)	-	2.19	-	1.84		Agonist Mixed CB_1_/CB_2_	[56,57]
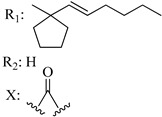 **203**	-	1.23	-	5.25	7.02	-	[56,57]
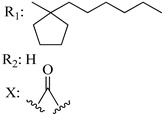 **204**	-	1.76	-	0.97	3.34	-	[56,57]
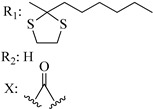 **205**	-	6.57	-	42.3	32.6	-	[56,57]
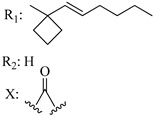 **206**	-	1.13	-	12.0	15.1	-	[56,57]
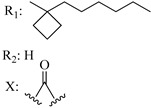 **207**	-	0.84	-	13.7	11.9	-	[56,57]
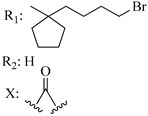 **208**	-	13.1	-	13.9	-	-	[56,57]
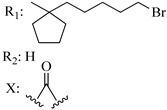 **209**	-	1.03	-	2.59	1.32	-	[56,57]
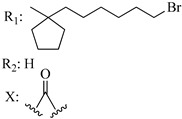 **210**	-	4.96	-	1.60	3.02	-	[56,57]
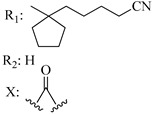 **211**	-	3.14		2.78	-	-	[56,57]
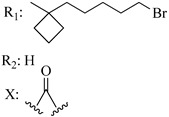 **212**	-	2.33		7.56	-	-	[56,57]
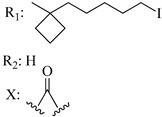 **213**	-	2.11		6.18	-	-	[56,57]

**Table 6:** Affinities (K*i*) of 7-(adamantan-1-yl)-2,2-dimethylchroman-5-ol analogs for rCB1, mCB2, and hCB2.

**Adamantyl Cannabinoid:**				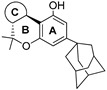			
**Compound**	**K*i* (nM)**					**Function**	**References**
	**rCB_1_**	**hCB_1_**	**rCB_2_**	**mCB_2_**	**hCB_2_**		
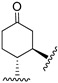 **214**	175.6	-	-	249.5	338	-	[107,108]
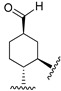 **215**	52.9	-	-	25.7	5.5	Agonist	[107,108]
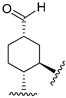 **216**	480.2	-	-	200.1	90.0	-	[107,108]
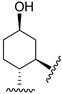 **217**	23.9	-	-	39.4	40.5	Agonist	[107,108]
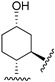 **218**	146.3	-	-	255.0	671.8	-	[107,108]
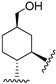 **219**	4.9	-	-	12.1	11.3	Agonist	[107,108]
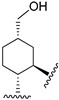 **220**	90.1	-	-	95.1	121.2	Agonist	[83,84]
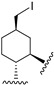 **221**	241.0	-	-	345.0	261.7	-	[83,84]
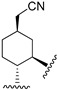 **222**	48.7	-	-	87.0	100.3	Agonist	[83,84]
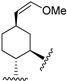 **223**	31.0	-	-	90.3	67.2	Agonist	[83,84]
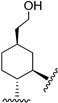 **224**	4.6	-	-	18.4	13.3	Agonist	[83,84]
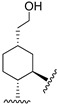 **225**	40.9	-	-	21	365.3	Agonist	[83,84]
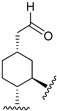 **226**	170.5	-	-	80.1	70.8	Agonist	[83,84]
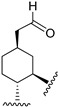 **227**	13.2	-	-	34.3	11.2	Agonist	[83,84]

### 7.6. Seven-Membered Lactone and Quinone in the Terpene Region

Incorporating a seven-membered lactone in ring C of the HHC scaffold generates a selective rCB1 agonist compound (**99a**, Table 7). It is interesting that its regioisomer (**99b**) did not display selectivity for rCB1 receptors. This confirms that the spatial configuration of the diastereomers plays a crucial role in the interactions with CB1 and CB2 receptors. Based on these results, we would propose the study of the affinities of a six-membered cannabinoid lactone for cannabinoid receptors.

Cannabinoid-receptor binding affinities presented in Table 8 demonstrated that the introduction of the 1,4-quinone moiety in ring C (compounds **139** and **140**) led to the loss of affinity towards cannabinoid receptors CB1 and CB2.

### 7.7. Nonclassical, Bicyclic-Hydrogenated Cannabinoids

Nonclassical, bicyclic-hydrogenated cannabinoids are exemplified by the paradigm compound CP-55,940 (**228**, Table 9). This compound acts as a full agonist for both CB1 and CB2 receptors. Compound **229** is obtained by removing the SAH chain from **228** and this leads to the reduction in affinity towards both receptors, CB1 and CB2. Attaching a cyclohexyl group to ring C increases the receptor binding affinity depending on the stereochemistry of the linkage of this group (compound **230** and **231**, Table 9).

### 7.8. Docking Studies and In Vitro Binding Affinities of HHC

Aviz-Amador [111] determined via molecular docking experiments in silico that HHC (compounds **1** and **7**, Table 10), Δ9-THC (**35b**, Table 10), and Δ8-THC (**36b**, Table 10) exhibit comparable high calculated binding energies to the CB2 receptor, although the binding energy of the *S*-HHC epimer (**7**) was a little lower. The hydrophobic interactions with the amino acid residues of the receptor protein are crucial and they led to equal results for the three cannabinoids. However, for the CB1 receptor, *R*-HHC (**1**) and Δ9-THC (**35b**) displayed similar high calculated binding affinities, while Δ8-THC (**36b**) and *S*-HHC (**7**) bound to this receptor with lower affinity. HHCs (**1** and **7**) exhibited partial CB1 and CB2 receptor agonist activity similar to Δ9-THC (**35b**). However, epimer **1** (*R*-HHC) binds with better affinity (K*i* = 15 and 13 nM at CB1 and CB2, respectively) than epimer **7** (*S*-HHC).

Thapa and co-workers [31,112,113] demonstrated that compounds **232** and **233** are potent angiogenesis inhibitors. They inhibit endothelial and tumor cell growth and lock the secretion of VEGF in cancer cells. Interestingly, these two compounds have poor binding affinities for CB1 and CB2 receptors, showing lower binding energy for both receptors.

Theses in in vitro and in silico studies related to binding affinities of HHC analogs prove how minimal alterations to the HHC scaffold can lead to notable differences in the biological activity of these compounds. Additionally, these results evidence the importance to isolate or of a single diastereomer to study how influential the changes are in the three-dimensional structure regarding both toxicology and potency.

## 8. Pharmacological and Toxicological Properties of Saturated Cannabinoids

Given the emergence of in vivo studies on the use of saturated cannabinoids in the treatment of various diseases, including cancer [15,114,115,116,117,118], neurological disorders [64,119,120], and diabetes [121,122], but also the prevalence of the consumption of these compounds [28], there is a crucial need to better comprehend their pharmacology and toxicology. In particular, the role of intrinsic efficacy in abuse-related effects, major metabolites, and adverse effects should be the subject of future study. Very limited information is available on the safety of saturated cannabinoids in humans, and serious health damage is highly likely to occur in those who abuse them. In particular, such information will help public health understanding of the adverse effect profile that differs from saturated cannabinoids to marijuana [123].

### 8.1. In Vitro Effects of Saturated Cannabinoid Analogs in Pancreatic Cell Lines

We recently reported the preliminary outcomes of the anticancer properties of HHC analogs in four pancreatic cancer cell lines: PANC-1, HPAF-II, AsPc-1, and MIA-PaCa2 [124,125]. Both the (*R*)-HHC and (*S*)-HHC epimers equally reduced the proliferation of cancer cells with IC_50_ values extending from 10.3 to 27.2 μM. These values are similar to the IC_50_ values of the anticancer agents olaparib or veliparib, resulting in more efficient compounds for the specific treatment of pancreatic cancer. Optimization led to novel saturated cannabinoids with greater cytotoxicity towards comparable cell lines [125]. The CCL compounds that were obtained for Colorado Chromatography Lab have exhibited 400–900 nm values against MiaPaCa-2 and PANC-1 cell lines, being over an order of magnitude more potent than Gemcitabine [126]. Although the IC_50_ values are lower compared to other active antineoplastic compounds on the market, the treatment of pancreatic cancer is still evolving and the need to produce antineoplastics is pertinent. Continued SAR and analog studies are currently being conducted for our research group to increase bioavailability and increase IC_50_ values to lower nanomolar concentrations, with future results potentially supporting our experimental claims.

### 8.2. In Vivo Effects of Saturated Cannabinoid Analogs

CBD and THC have been extensively studied and many in vivo studies related to their anticancer and nausea and pain-relieving activity have been carried out. There are even several FDA-approved human treatments. However, there are very few in vivo studies using saturated cannabinoids and only nabilone (**88**) has been approved by the FDA to treat nausea and vomiting caused by cancer chemotherapy [127]. Also, preliminary studies propose that nabilone can be used as an acceptable treatment option for severe behavioral problems in adults with intellectual and developmental disabilities [128].

Our research group conducted in vivo studies with CCL compounds to prove the pre-clinical efficacy of these saturated cannabinoids in a subcutaneous xenograft of pancreatic ductal adenocarcinoma cell lines [126]. These studies indicate that CCL compounds slow down the development of human tumors in a mouse subcutaneous xenograft model, and most intriguingly, demonstrated ~50% tumor growth inhibition without significant body weight loss or any unusual signs of toxicity via the oral route (31 mg/kg).

The new and rediscovered cannabinoids have no pre-clinical safety profile performed on them and are being consumed. We executed a pre-clinical assessment on the racemic mixture of HHC [11] and H_4_CBD [129] to provide a preclinical assessment profile for the consumption of these compounds. The analysis of the different cell types revealed varying responses to H_4_CBD and HHC. Lung fibroblasts (NHLF) showed a concentration-dependent reduction in cell viability, with maintained concentrations over 24 h at 6.25–30 μM ensuing in a significant loss of viability. On the contrary, hepatocytes showed a trend of reduced viability at longer exposure times and higher concentrations, but severe cytotoxicity was not observed. This suggests that hepatocytes are less susceptible to the cytotoxic effects of H_4_CBD and HHC compared to NHLF. In the hERG assay, H_4_CBD and HHC did not inhibit the action potentials within cardiomyocytes, indicating no inhibition of ion channels involved in cardiac function.

These findings provide insight into the cytotoxic effects of H_4_CBD and HHC and contribute to establishing research and safety parameters as these compounds continue to gain attention.

Cannazza and coworkers [130] led some in vivo behavioral tests on mice to evaluate the cannabimimetic activity of both HHC diastereomers. These tests judge spontaneous activity, catalepsy, analgesia, and changes in rectal temperature, which are physiological symptoms of THC activity. The outcomes revealed that compound **1** (9*R*-HHC) extensively altered spontaneous locomotion and pain relief while compound **7** (9*S*-HHC) had insignificant activity. These discoveries support the in vitro results related to binding affinity to CB1 and CB2 receptors of both diastereomers.

Graziano et al. [14] carried out studies in vivo with both HHC diastereomers displaying effects in the central nervous system, with lower potency than Δ9-THC. Also, this study revealed that 9(*R*)-HHC is more potent than 9(*S*)-HHC, suggesting that this diastereomer could lead to a possible addiction potential.

## 9. Summary and Outlook

The markets for hydrogenated cannabinoids and related synthetic cannabinoids are rapidly evolving areas with relatively limited information currently available. This review summarizes the discovery, novel synthetic pathways, and pharmacology studies of classical, non-classical, and hybrid hydrogenated cannabinoids, discussing the most critical point of view in this area. This is harmonized with a summary and comparison of the cannabinoid receptor affinities of various classical, hybrid, and non-classical saturated cannabinoids. A discussion of structure–activity relationships with the four different pharmacophores found in the cannabinoid scaffold is added to this review.

Saturated cannabinoid-based therapies like nabilone suffer from undesirable pharmacological properties including poor bioavailability, the unpredictable onset/offset of action, and detoxification. The clear medical need for novel cannabinoid-based medications has encouraged us to pursue this review. We believe the design and development of novel hydrogenated cannabinoids should address the quest for new selective antagonist-based cannabinoids for CB2 receptors with improved drug ability, i.e., improved oral availability, a predictable time course of action, and controllable detoxification. The design of new CB2-selective hydrogenated THC analogs should have little or no affinity for the CB1 receptor, thus eliminating the risk of central CB1-mediated psychotropic effects.

Furthermore, the input of an azido, isothiocyanate, and cyano-moiety at diverse tactical positions within these nonclassical–hybrid hydrogenated cannabinoids and the emergence of covalent bonds with different amino acid residues on the CB1 and CB2 receptors allow for a more comprehensive searching of the stereochemical features of the receptor active sites.

The cannabinoid-based research should focus on accomplishing more efficient enantioselective routes to furnish novel synthetic and highly enantiopure-saturated nonclassical and hybrid cannabinoids at the disposal of chemists. Many more exclusive ligands can be minded and explored for their pharmacological activity. The accessibility of the functionalized bi- and tricyclic cannabinoid skeleton will facilitate the scanning of the CB1 and CB2 receptors. A better comprehension of the receptor binding site may make it possible to project cannabinoids with controlled selectivity and affinity for CB1, CB2, or both cannabinoid receptors to potentially support in the selective handling of the endocannabinoid system.

The limitation of the study of saturated cannabinoids is that most of the articles do not offer a multiparty vision between the challenges of organic synthesis, medicinal chemistry, and toxicology of these compounds, which play an important role in the cannabinoid research.

## Figures and Tables

**Table 1:** Results of the reactions of resorcinols and naphthols with citronellal ^a^.

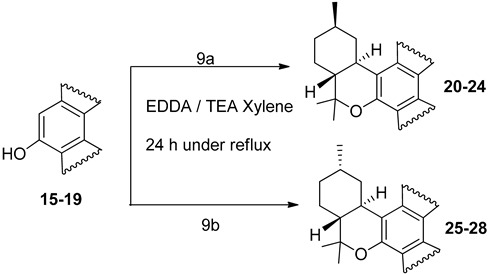				
**Entry**	**Starting Material**	**Citronellal**	**Product**	**Yield (%)**
1	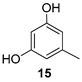	**9a**	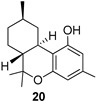	68
2	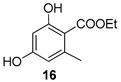	**9a**	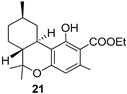	87
3	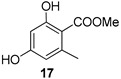	**9a**	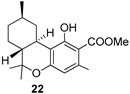	75
3	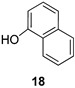	**9a**	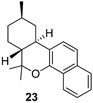	92
4	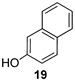	**9a**	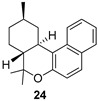	72
5	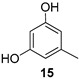	**9b**	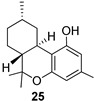	70
6	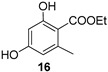	**9b**	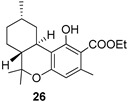	87
7	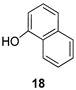	**9b**	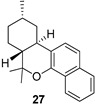	90
8	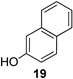	**9b**	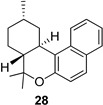	75

^a^ Reaction conditions: starting material (1.0 mmol), citronellal (1.5 mmol), EDDA (20% mol), TEA (0.2% mol) in xylene [21].

**Table 2:** Affinities (K*i*) of hydrogenated CBD analogs for rCB1 and mCB2.

Compound	K*i* (nM)					Function	References
	rCB_1_	hCB_1_	rCB_2_	mCB_2_	hCB_2_		
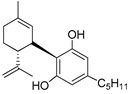 **34b**	>10,000	-		>10,000	-	-	[63]
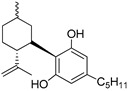 **102a**	>1000	-	-	-	-	-	[9,63]
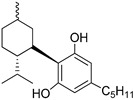 **103a**	145	-	-	-	-	-	[9,63]
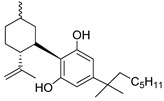 **102b**	124	-	-	-	-	-	[9,63]
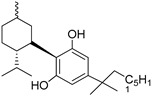 **103b**	17	-	-	-	-	-	[9,63]

**Table 3:** CB1/CB2 cannabinoid receptor binding affinity for machaeriol, machaeridiol, and their homologs.

Compound	K*i* (μM)					Function	References
	rCB_1_	hCB_1_	rCB_2_	mCB_2_	hCB_2_		
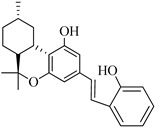 **39** (Machaeriol C)	3.27	-	7.76	-	-	-	[103]
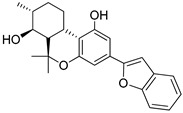 **43** (Machaeriol D)	1.75	-	1.30	-	-	-	[103]
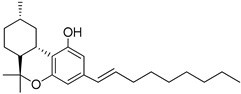 **165**	0.34	-	0.57	-	-	-	[103]
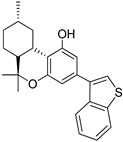 **166**	>1000	-	0.040	-	-	CB2 selective agonists	[103]
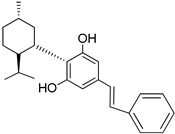 **106** (Machaeridiol A)	>1000	-	1.77	-	-	CB2 selective agonists	[103]
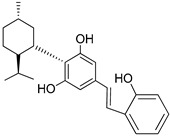 **167** (Machaeridiol B)	>1000	-	2.18	-	-	CB2 selective agonists	[103]
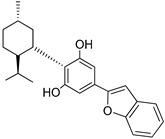 **168** (Machaeridiol C)	>1000	-	1.11	-	-	CB2 selective agonists	[103]

**Table 7:** Affinities (K*i*) of Cannabinoid Lactones for rCB1, mCB2, and hCB2.

Compound	K*i* (nM)					Function	References
	rCB_1_	hCB_1_	rCB_2_	mCB_2_	hCB_2_		
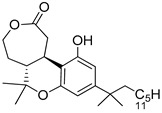 **99b**	99.0 ± 11	-	-	803.0 ± 87	94.1 ± 13	-	[58]
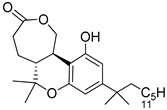 **99a**	4.6 ± 2.8	-	-	792.3 ± 76	54.1 ± 7	Agonist CB_1_	[58]

**Table 8:** Affinities (K*i*) of Cannabinoid–quinone for rCB1 and mCB2.

Compound	K*i* (nM)					Function	References
	rCB_1_	hCB_1_	rCB_2_	mCB_2_	hCB_2_		
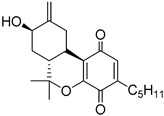 **140**	919.7	-	-	2034.1	-	-	[78]
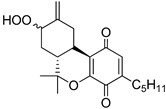 **139**	286.4	-	-	464	-	-	[78]

**Table 9:** Affinities (K*i*) of non-classical cannabinoids for hCB1 and hCB2.

Compound	K*i* (nM)					Function	References
	rCB_1_	hCB_1_	rCB_2_	mCB_2_	hCB_2_		
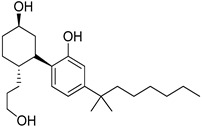 **228 (CP-55,940)**	-	0.58	-	-	0.69	Agonist	[109,110]
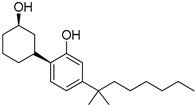 **229**	-	61.6	-	-	91.0	-	[109,110]
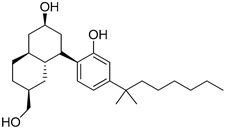 **230**	-	1.0	-	-	2.4	-	[11,110]
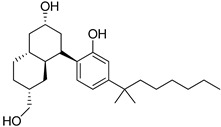 **231**		7079	-	-	7585	-	[109,110]

**Table 10:** Molecular Docking with D9THC (**35b**), D8THC (**36b**), HHC (**1** and **7**), and HHC analogs (**232** and **233**) binding with CB1 and CB2 receptor.

Compound	Binding Energy (kcal/mol)		Interaction Type	Ki (nM)		References
	CB_1_	CB_2_		hCB_1_	hCB_2_	
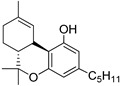 **35b**	−9.4	−10.4	**CB_1_**: Alkyl, π-alkyl, π–σ bond, C–H bond, van der Waals. **CB_2_**: Alkyl, π-alkyl, π–π-T-shaped, π–σ bond	15	9.1	[91,111]
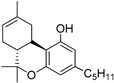 **36b**	−6.9	−10.1	**CB_2_**: Alkyl, π-alkyl, π–π-T-shaped, π-Donor-H bond	440	337	[91,111]
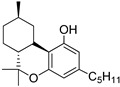 **1**	−9.1	−10.3	**CB_2_**: Alkyl, π-alkyl, π–π-T-shaped, π–σ bond	15	13	[91,111]
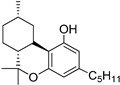 **7**	−7.2	−9.1	**CB_2_**: Alkyl, π-alkyl, π–π-T-shaped, π–π-Stacked	176	105	[91,111]
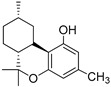 **232**	−6.4	−7.1	-	>1000	>100	[31,112,113]
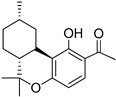 **233**	−5.9	−6.5	-	>1000	>100	[31,112,113]

## Data Availability

No new data were created or analyzed in this study. Data sharing is not applicable to this article.
